# A substitution mutation in a conserved domain of mammalian acetate-dependent acetyl CoA synthetase 2 results in destabilized protein and impaired HIF-2 signaling

**DOI:** 10.1371/journal.pone.0225105

**Published:** 2019-11-14

**Authors:** Jason S. Nagati, Min Xu, Trent Garcia, Sarah A. Comerford, Robert E. Hammer, Joseph A. Garcia

**Affiliations:** 1 Department of Medicine, Columbia University Medical Center, New York, New York, United States of America; 2 Department of Pathology, University of Texas Southwestern Medical Center, Dallas, Texas, United States of America; 3 Department of Molecular Genetics, University of Texas Southwestern Medical Center, Dallas, Texas, United States of America; 4 Department of Biochemistry, University of Texas Southwestern Medical Center, Dallas, Texas, United States of America; 5 Department of Research, James J. Peters VA Medical Center, New York, New York, United States of America; Ludwig-Maximilians-Universitat Munchen Adolf-Butenandt-Institut, GERMANY

## Abstract

The response to environmental stresses by eukaryotic organisms includes activation of protective biological mechanisms, orchestrated in part by transcriptional regulators. The tri-member Hypoxia Inducible Factor (HIF) family of DNA-binding transcription factors include HIF-2, which is activated under conditions of oxygen or glucose deprivation. Although oxygen-dependent protein degradation is a key mechanism by which HIF-1 and HIF-2 activity is regulated, HIF-2 is also influenced substantially by the coupled action of acetylation and deacetylation. The acetylation/deacetylation process that HIF-2 undergoes employs a specific acetyltransferase and deacetylase. Likewise, the supply of the acetyl donor, acetyl CoA, used for HIF-2 acetylation originates from a specific acetyl CoA generator, acetate-dependent acetyl CoA synthetase 2 (Acss2). Although Acss2 is predominantly cytosolic, a subset of the Acss2 cellular pool is enriched in the nucleus following oxygen or glucose deprivation. Prevention of nuclear localization by a directed mutation in a putative nuclear localization signal in Acss2 abrogates HIF-2 acetylation and blunts HIF-2 dependent signaling as well as flank tumor growth for knockdown/rescue cancer cells expressing ectopic Acss2. In this study, we report generation of a novel mouse strain using CRISPR/Cas9 mutagenesis that express this mutant Acss2 allele in the mouse germline. The homozygous mutant mice have impaired induction of the canonical HIF-2 target gene erythropoietin and blunted recovery from acute anemia. Surprisingly, Acss2 protein levels are dramatically reduced in these mutant mice. Functional studies investigating the basis for this phenotype reveal multiple protein instability domains in the Acss2 carboxy terminus. The findings described herein may be of relevance in the regulation of native Acss2 protein as well as for humans carrying missense mutations in these domains.

## Introduction

Coordinate regulation of cellular biological processes is facilitated by signaling mechanisms that bridge intracellular compartments. In eukaryotes, signal transducers integral to these signaling mechanisms include cytosolic proteins with additional roles in other subcellular compartments. Acetylation of histone and non-histone proteins can affect nuclear gene expression. Because acetylation employs acetyl groups derived from acetyl CoA, regulated production of *de novo* acetyl CoA may be an important means by which changes in metabolic status alter nuclear gene expression patterns.

Acetate-dependent acetyl CoA synthetase 2 (Acss2) is a cytosolic acetyl CoA generator identified on the basis of its homology to mitochondrial localized acetate-dependent acetyl CoA synthetase 1 (Acss1)[1]. Genetic ablation of Acss2 in mice revealed a role in nuclear signaling mediated by the stress-responsive transcription factor Hypoxia Inducible Factor 2 (HIF-2)[2], which undergoes cyclical acetylation/deacetylation modifications by the acetyl transferase Creb binding protein (Cbp) and the deacetylase Sirtuin 1 (Sirt1), respectively, when transcriptionally active[3, 4]. We observed that Acss2 protein is enriched in the nucleus with environmental stress[5], which was confirmed by other investigators[6, 7]. Knockdown/rescue cell lines expressing HIF-2 with substitutions for lysine residues modified by acetylation phenocopied knockdown/rescue cells expressing mutant Acss2[5, 8]. We postulated that Acss2 generates a nuclear acetyl CoA pool used selectively by Cbp to acetylate HIF-2 and may further impact nuclear gene expression through modification of histone proteins[6, 7, 9–11].

Mice carrying null mutations in the Acss2 gene exhibited impaired HIF-2 dependent molecular and physiological responses[2]. Stably transformed knockdown/rescue cells expressing a mutant Acss2 protein with acidic amino acid substitutions for basic amino acid residues in a putative nuclear localization signal that resulted in cytosol-restriction, hereby referred to as ED Acss2 protein, displayed impaired HIF-2 dependent signaling and a blunted ability for flank tumor growth[5, 8]. We were interested in developing a mouse model expressing ED Acss2 instead of endogenous wild-type (WT) Acss2 to investigate the role of nuclear Acss2 in normal mammalian physiology. Using CRISPR/Cas9 targeted mutagenesis, we introduced substitution mutations in the endogenous Acss2 locus in mice. Mice homozygous for the ED mutation are viable and fertile. In this study, we report the effects of the ED substitution mutation on Acss2 function as well as on HIF-2 dependent molecular and physiological responses to acute hemolytic anemia.

## Results

**Fig 1:**
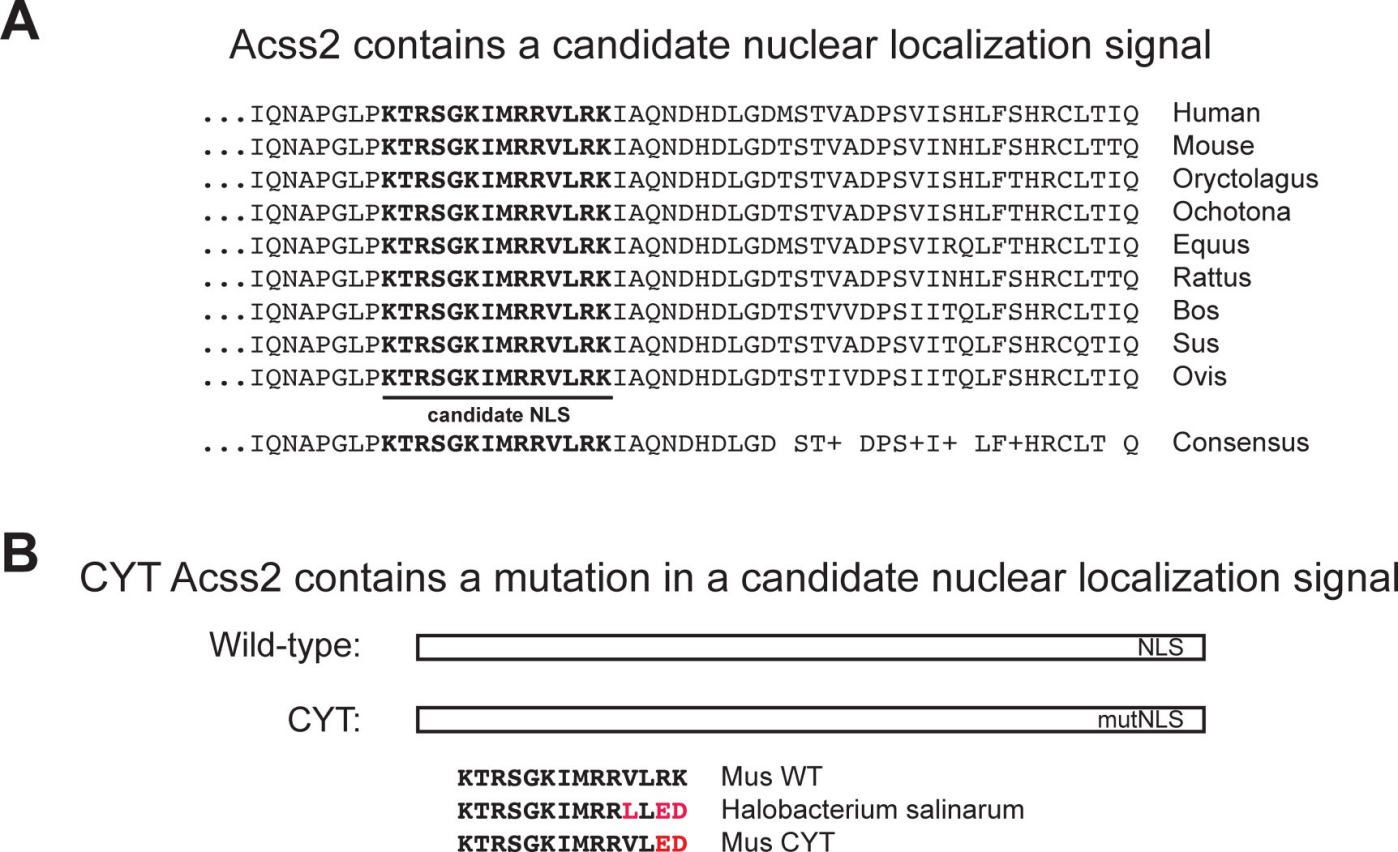
Acss2 contains a conserved candidate nuclear localization signal. (A) Multiple alignments of diverse mammalian Acss2 reveal strong conservation of a basic domain in the Acss2 carboxy terminal region proposed to function as a candidate nuclear localization signal (NLS). (B) The prokaryotic Acss2 homolog in *Halobacterium salinarum* contains acidic residues instead of basic residues found in mammalian Acss2 proteins, which are the basis for the ED substitution mutation.

We first performed a homology assessment of mammalian Acss2 to identify conserved domains present in a region of Acss2 containing a basic residue-rich domain implicated in nuclear localization. The result of multiple alignments revealed a highly conserved amino acid domain encompassing the basic region (Fig 1A). The substitution mutations used to generate ED Acss2, based on their position in a prokaryotic Acss2 homolog[12], are present in this conserved domain (Fig 1B). We employed CRISPR/Cas9 to knock-in the ED mutation in mice. The resultant founder mice were back-crossed into wild-type C57BL/6J mice for several generations to reduce off-target effects of the CRISPR/Cas9 mutagenesis process.

**Fig 2:**
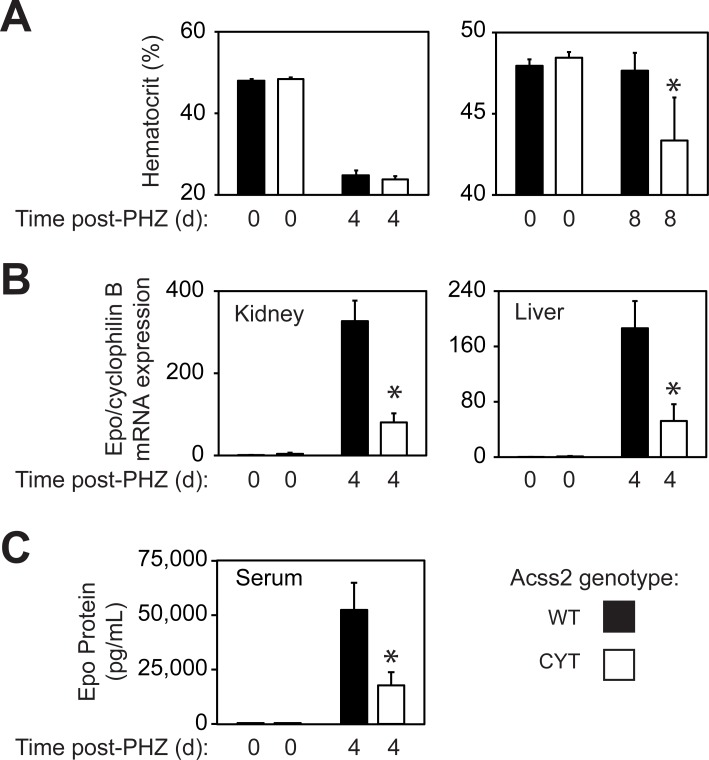
ED Acss2 mice have impaired physiological and molecular responses. (A) WT and ED mice have similar baseline hematocrit levels. Following phenylhydrazine (PHZ) exposure, WT and ED mice were selected for a comparable degree of anemia. At 8 days following PHZ exposure, ED mice had a blunted recovery in hematocrit levels relative to WT mice (mean with SEM, n = 7 mice per group, **P = 0.08). (B) Induction of the HIF-2 target gene erythropoietin (Epo) is impaired in kidney (mean with SEM, n = 8 mice per group, *P = 0.018) and liver (mean with SEM, n = 8 mice per group, *P = 0.013) of anemic ED mice versus WT mice at 4 days following PHZ exposure. (C) Serum Epo protein levels are also reduced in ED mice relative to WT mice (mean with SEM, n = 8 mice per group, *P = 0.017).

Heterozygous ED Acss2 mating pairs generated homozygous Acss2 mice expressing only ED Acss2 protein, hereafter referred to as ED mice, and wild-type (WT) litter mates. WT and ED mice exhibited no significant differences in baseline hematocrit designated as day 0. These mice were then injected with phenylhydrazine (PHZ) at 4 weeks of age to induce an acute hemolytic anemia. Hematocrits obtained at 4 days after PHZ injection, the nadir of the acute hemolytic anemia, identified mice with comparable severe anemia (20–25%) in both WT and ED groups. Hematocrits obtained 8 days after PHZ injection for WT mice revealed resolution of the acute anemia in the recovery phase, whereas ED mice lacked this response (Fig 2A). To determine the effect of the ED mutation on induction of erythropoietin (Epo), a canonical HIF-2 target gene, we measured Epo mRNA levels in kidney and liver obtained from WT and ED mice at 4 days following PHZ injection. The ED mice exhibited a blunted induction of Epo in kidney and liver, the predominant sites of endocrine Epo induction, (Fig 2B) and also had reduced serum Epo protein levels relative to WT controls (Fig 2C).

**Fig 3:**
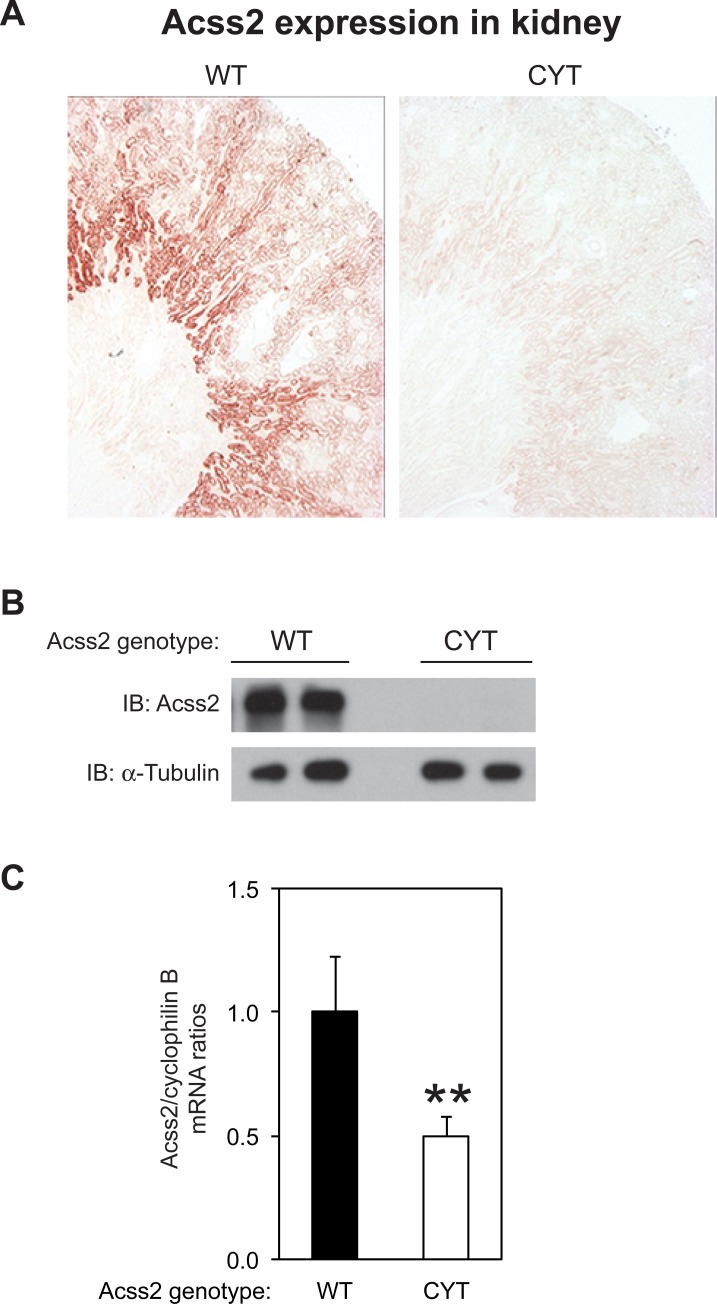
Acss2 protein levels are markedly reduced in ED Acss2 kidney. (A) Immunohistochemical and (B) immunoblot assays (two independent samples per group; normalized to protein amount and compared to immunoblot of α-tubulin) reveal virtually absent staining of Acss2 protein in kidney of ED Acss2 mice. (C) Acss2 mRNA levels in ED kidneys are reduced to approximately 50% of levels found in WT mice (mean with SEM, n = 8 mice per group, **P = 0.062).

**Fig 4:**
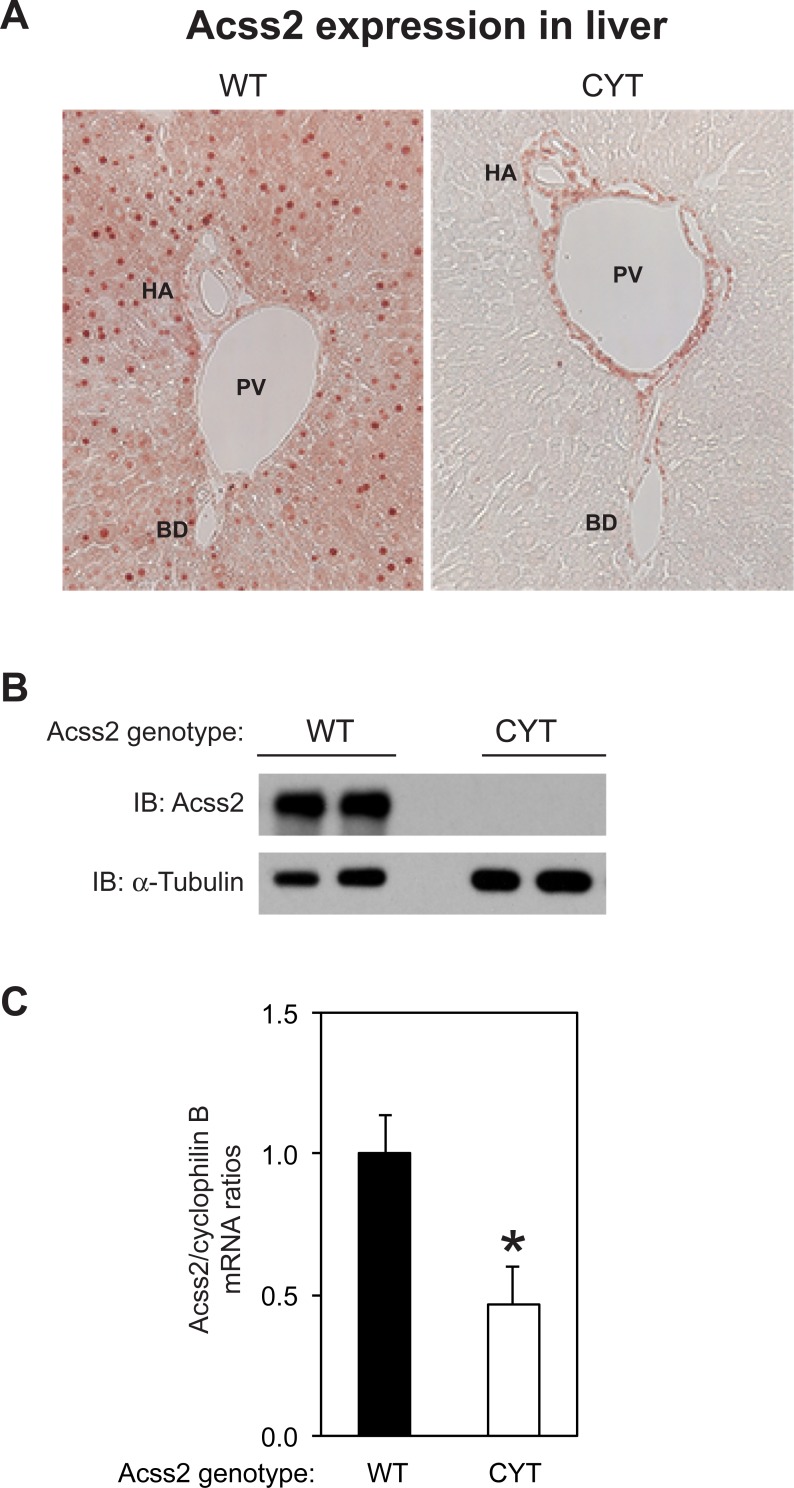
Acss2 protein levels are markedly reduced in ED Acss2 liver. (A) Immunohistochemical and (B) immunoblot assays (two independent samples per group; normalized to protein amount and compared to immunoblot of α-tubulin) reveal reduced staining of Acss2 protein in liver of ED Acss2 mice with some immunoreactivity noted around the portal vein (PV), hepatic artery (HA), and bile duct (BD). (C) Acss2 mRNA levels in ED liver are reduced to approximately 50% of levels found in WT mice (mean with SEM, n = 8 mice per group, *P = 0.016).

We next asked whether the ED mutation affects Acss2 protein characteristics in the kidney (Fig 3). Using immunohistochemical localization, we noted that Acss2 protein levels were markedly reduced in ED kidney. These results were confirmed by immunoblot analyses and could not be explained solely by a reduction in Acss2 mRNA levels. Similar results were observed in liver samples from ED mice except that some immunoreactivity was observed in liver, particularly around the portal vein, hepatic artery, and, to a lesser degree, bile duct (Fig 4).

**Fig 5:**
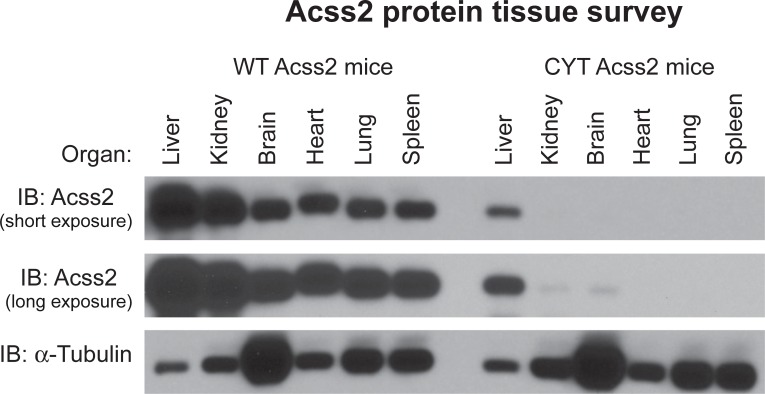
Acss2 protein levels are globally reduced in ED Acss2 mice. Tissue survey of ED mice reveals global reduction or absence of Acss2 protein in liver, kidney, brain, heart, lung, and spleen. Short and long exposures of Acss2 protein immunoreactivity are shown normalized to protein amount and compared to immunoblot of α-tubulin.

**Fig 6:**
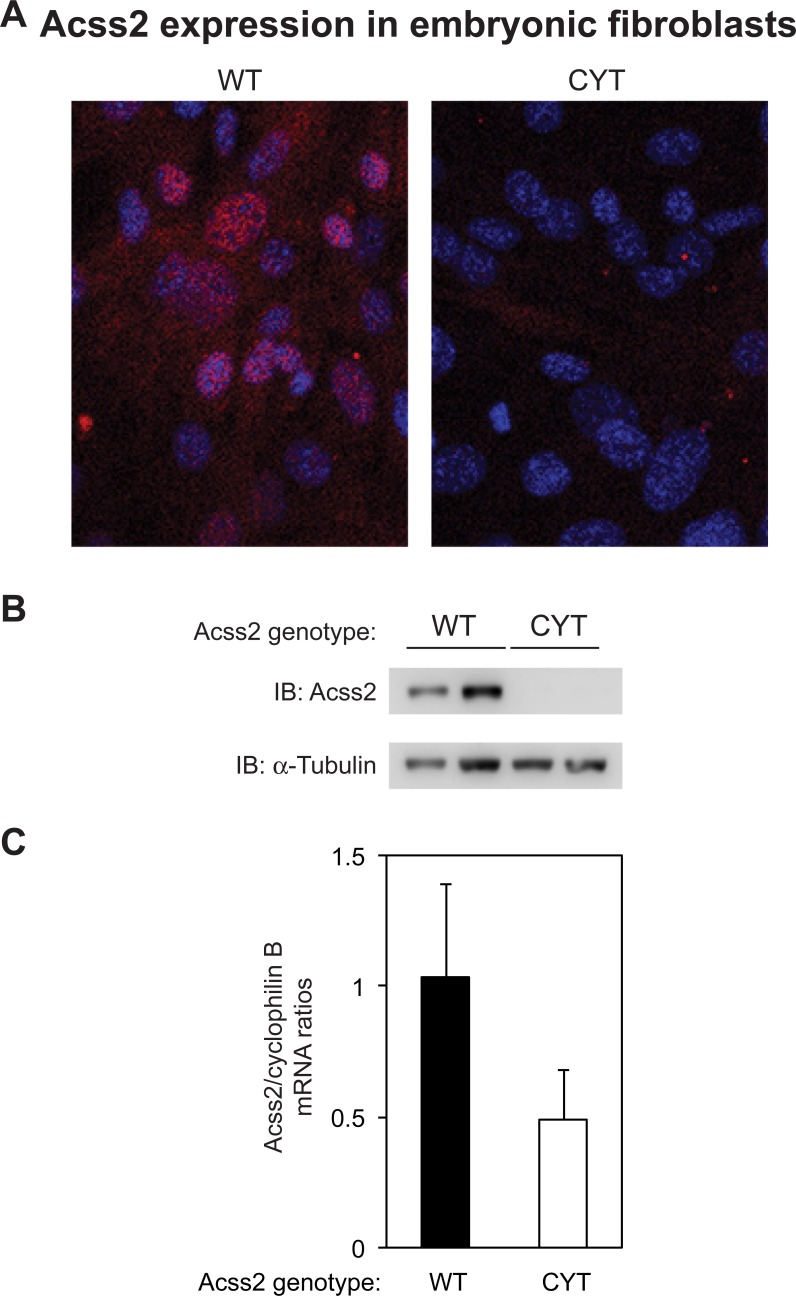
Acss2 protein levels are markedly reduced in ED Acss2 mouse embryonic fibroblasts. (A) Immunofluorescent and (B) immunoblot analyses of mouse embryonic fibroblasts (MEF) (two independently derived cell lines; normalized to protein amount and compared to immunoblot of α-tubulin) derived from ED Acss2 pups reveal undetectable Acss2 protein levels. Acss2 immunoreactivity is noted in the cytosol as well as nucleus of WT MEF as evident by colocalization of Acss2 signal (red) with nuclei stained with DAPI (blue). (C) Acss2 mRNA levels in ED MEF are not significantly reduced relative to Acss2 mRNA levels in WT MEF (n = 3 per group, P = 0.259).

To determine whether Acss2 protein levels were globally reduced, we surveyed a number of tissues from adult ED mice for Acss2 protein levels relative to tissues derived from WT mice by immunoblot analysis (Fig 5). Markedly reduced Acss2 protein levels were observed in liver with short exposures and barely detectable levels of Acss2 protein were noted in kidney and brain of ED mice with long exposures. Also of note, Acss2 protein obtained from heart of adult WT mice migrated at a slightly higher apparent molecular weight than Acss2 protein obtained from other tissues examined. We prepared mouse embryonic fibroblasts (MEF) from ED embryos[13]. ED MEF exhibited reduced Acss2 protein expression relative to MEF derived from WT embryos and Acss2 mRNA levels in ED MEF also trended lower, although these differences did not reach statistically significant levels (Fig 6).

The reduced level of Acss2 protein in ED mice suggested that the effect of the ED mutation was predominantly on Acss2 protein stability. One possible explanation is the presence of destabilization domains in Acss2 that are unmasked as a result of the ED mutation. Molecular modeling and structural studies of prokaryotic Acss2 homologues indicated the likely presence of multiple organized and unorganized structural features in the Acss2 superfamily[14]. These features reside in two large globular regions, the first comprising approximately the amino 80% of Acss2 and the second encompassing the carboxy 20% regions of Acss2 referred to as the hinge region.

**Fig 7:**
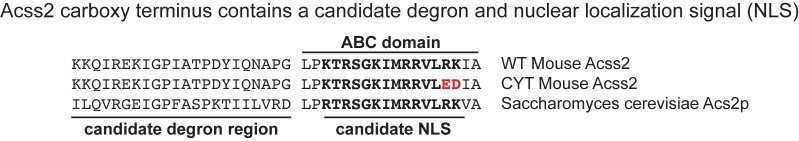
The Acss2 hinge region contains multiple candidate functional domains. Alignment of mouse WT Acss2 protein, mouse ED Acss2 protein, and *Saccharomyces cerevisiae* Acss2 homolog Acs2p protein reveals a candidate degron region as well as a candidate nuclear localization signal (NLS) encompassed in the Acss2 basic residue-rich conserved domain (ABC domain).

The hinge region contains several domains involved in enzymatic function as well as other elements of interest (Fig 7). One potentially important element is a region coincident with a putative degron identified in a yeast Acss2 homolog[15], which encompasses the alpha 18 and A9 domains. A second element of interest is a highly conserved basic domain constituting a candidate nuclear localization signal (NLS), which we refer to as the Acss2 basic-rich conserved domain (ABC domain).

**Fig 8:**
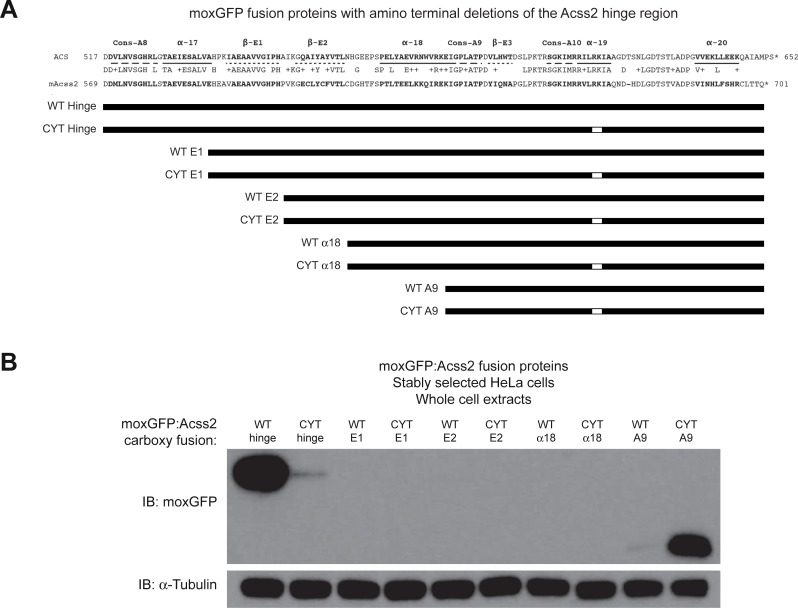
Truncating the Acss2 hinge region results in protein destabilization of a fusion protein. (A) Stable HeLa cell lines expressing amino terminal truncations of the WT or ED Acss2 carboxy terminal hinge region fused to the carboxy terminus of the green fluorescent protein moxGFP were generated by lentiviral transduction followed by drug selection. (B) Immunoblots for the moxGFP:Acss2 fusion proteins were performed to assess relative protein levels (normalized to protein amount and compared to immunoblot of α-tubulin).

To assess for the presence of destabilization domains, we designed lentiviral expression vectors that encoded the hinge region of Acss2 fused to the carboxy terminus of the green fluorescent protein moxGFP[16]. We then generated additional expression constructs encoding fusion proteins with amino truncations of Acss2 protein (Fig 8A). For all constructs, we compared the WT version to a comparable version with the ED mutation. The fusion proteins were expressed in HeLa cells following stable selection and subjected to immunoblot analyses to assess protein stability (Fig 8B). Whereas moxGFP:WT hinge fusion protein was stable, moxGFP:ED hinge fusion protein was unstable. Amino deletion of the E1 and E2 domains in the hinge region destabilized both WT and ED moxGFP fusion proteins to undetectable levels. Interestingly, further amino truncations that eliminated the alpha 18 domain in the Acss2 hinge region, which partial deletes the candidate degron domain, resulted in slight stability of moxGFP:ED, but not moxGFP:WT, fusion protein.

**Fig 9:**
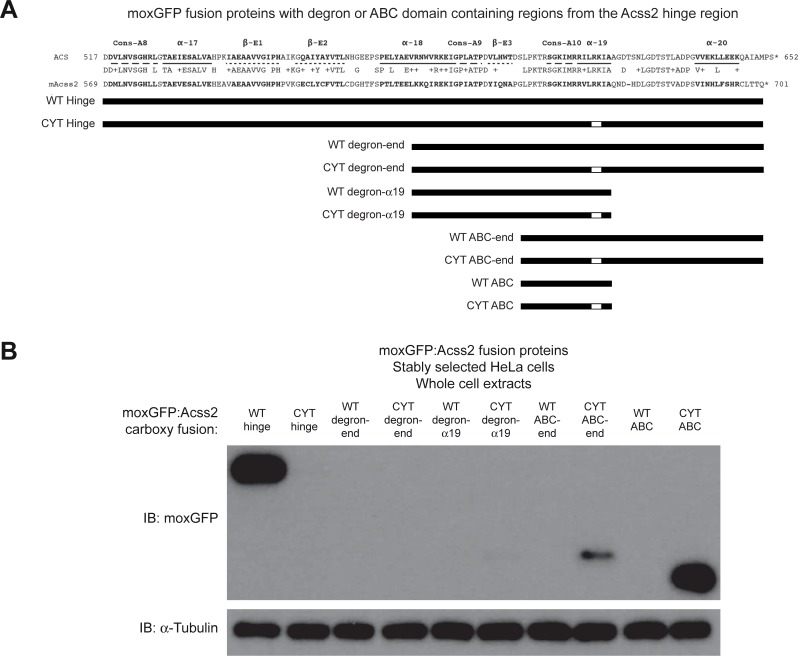
The Acss2 hinge region contains protein destabilization and masking elements. (A) Stable HeLa cell lines expressing select amino and carboxy terminal truncations as well as isolated sequences of the WT or ED Acss2 carboxy terminal hinge region fused to the carboxy terminus of the green fluorescent protein moxGFP were generated by lentiviral transduction followed by drug selection. (B) Immunoblots for the moxGFP:Acss2 fusion proteins were performed to assess relative protein levels (normalized to protein amount and compared to immunoblot of α-tubulin).

The increased stability of the moxGFP:ED fusion protein following amino truncation through the alpha 18 region suggested that the candidate degron may be a key determinant of protein stability. Using moxGFP:WT and moxGFP:ED fusion proteins as starting points, we next generated additional constructs with more precise amino and carboxy terminal deletions upstream and downstream of the ABC domain (Fig 9A). Immunoblot analyses revealed that an amino deletion of the parental WT or ED hinge constructs, which retained the candidate degron domain and downstream regions, was unstable and undetectable (Fig 9B). Additional deletion of sequences downstream of the ABC domain resulted in slight stability of moxGFP:ED, but not moxGFP:WT, fusion protein, suggesting the potential existence of a protein destabilization element in the Acss2 carboxy terminus. Similar to the alpha 18 domain amino terminal deletion constructs, an amino terminal deletion of the degron resulted in slightly greater stability of moxGFP:ED, but not moxGFP:WT, fusion protein.

**Fig 10:**
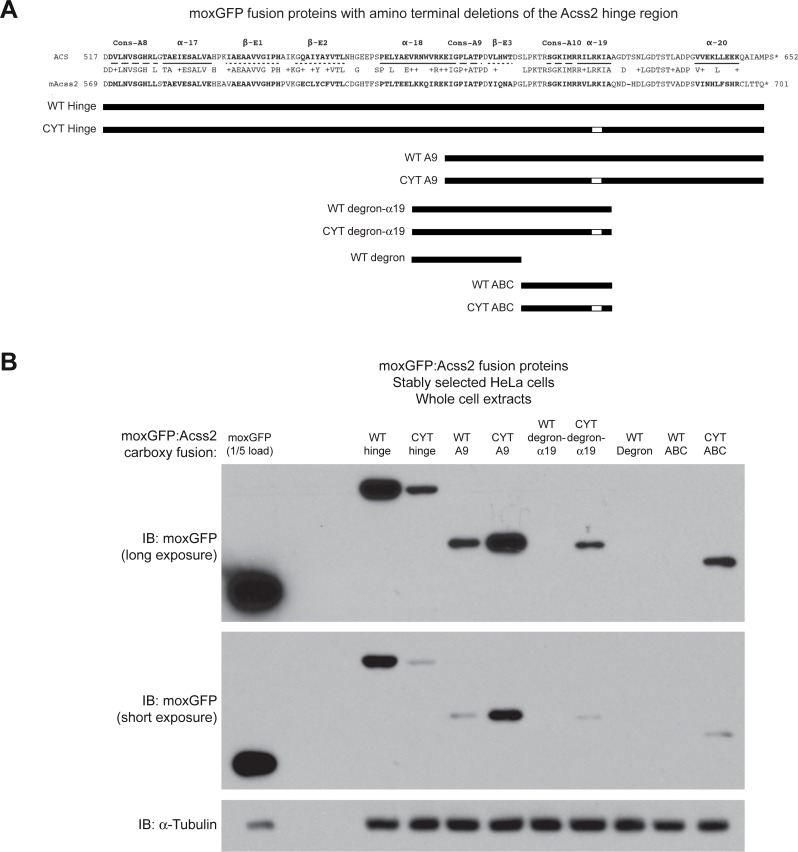
The isolated degron and ABC domains are potent protein destabilization elements. (A) Stable HeLa cell lines expressing select regions of the WT or ED Acss2 carboxy terminal hinge region fused to the carboxy terminus of the green fluorescent protein moxGFP were generated by lentiviral transduction followed by drug selection. (B) The moxGFP:Acss2 fusion proteins were examined by immunoblot to assess relative protein levels. Because the level of parental moxGFP fusion protein in a stable cell line was substantially higher than levels of all moxGFP:Acss2 fusion proteins examined, the amount of protein extract loaded from the moxGFP only stable cell line was one-fifth that of the stable moxGFP:Acss2 fusion protein cell lines as reflected in the reduced immunoblot signal for the comparison protein, α-tubulin.

The results from the amino and carboxy deletion moxGFP fusion protein constructs in summary indicate that the candidate degron region, ABC domain, and possibly other domains in Acss2 can function as potent protein destabilization elements. To directly compare the most informative constructs of this study, we generated independent cell lines and repeated the immunoblot experiments; we also examined the ability of the isolated degron region to function as a protein destabilization domain (Fig 10). The results demonstrate that moxGFP:WT hinge fusion protein is the most stable of the WT constructs whereas moxGFP:ED A9 is the most stable of the ED constructs. Notably, although the ED mutation in the context of the isolated ABC domain renders moxGFP:ED ABC detectable, its stability relative to either the ED A9 or WT hinge fusion constructs is reduced, especially when compared to the parental moxGFP fusion protein partner, which was loaded at one-fifth the amount of the moxGFP:Acss2 fusion protein samples. Finally, we note that the moxGFP:WT degron and moxGFP:WT ABC domain fusion proteins were undetectable even with prolonged exposures, consistent with an ability of these isolated domains to function as potent protein destabilization elements.

## Discussion

The results of this study indicate that the mouse Acss2 protein can be profoundly affected by amino acid substitutions. Specifically, the ED substitution mutation of two acidic amino acids for two basic amino acids in the ABC domain results in drastic reductions of Acss2 protein levels in all tissues examined from ED mutant mice. The effect of the ED mutation is cell autonomous as ED mouse embryonic fibroblasts also exhibit marked differences in Acss2 protein levels. We were surprised to find such a potent effect of the ED substitution mutation in mice, given that our earlier studies revealed minimal effects of this mutation in knockdown/rescue studies[8]. When stably expressed in transformed HT1080 cells using integrated lentiviral vectors, and with concurrent knockdown of endogenous Acss2 by shRNA expression, ectopic rescue WT and ED Acss2 protein levels were comparable. Upon examination in other stably transformed cell lines, we noted at most a modest reduction in expression of ectopic ED Acss2 relative to ectopic WT Acss2 protein. Whether the increased levels of ectopic ED Acss2 protein levels in knockdown/rescue transformed cells are due to the malignant nature of the cell, a stabilizing effect of residual endogenous WT Acss2 on ectopic ED Acss2, or a consequence of the ectopic expression itself remains to be determined.

Several observations made with the immunohistochemical and immunoblot analyses of Acss2 in this study are worth mentioning. ED Acss2 protein is evident in immunohistochemical and immunoblot analyses of liver samples obtained from homozygous ED Acss2 mice, albeit at substantially reduced levels compared to Acss2 protein levels present in liver of WT Acss2 mice. ED Acss2 protein expressed in the liver of homozygous ED Acss2 mice appears to be localized to endothelial cells lining the portal vein. It is possible that exposure of these cells to elevated levels of acetate present in the portal circulation are relevant to this observation. Alternatively, the prominent role of liver in acetate utilization may have some role in this observation. The kidney and brain are other significant consumers of acetate. With longer exposures of Acss2 immunoblots, ED Acss2 protein is evident at very low levels in these organs. However, whether these are associated findings or causal relationships remains to be determined. Finally, we note that WT Acss2 protein in heart consistently migrated at a slightly greater molecular weight than WT Acss2 protein obtained from other tissues examined. Whether this difference in apparent size is due to Acss2 translated from alternative splice variants or start sites in the heart, or whether it is due to differences in post-translational modifications of Acss2 in heart versus other tissue remains to be determined.

Our initial investigation into the molecular basis for the destabilizing effect of the ED substitution mutation revealed at least two potent destabilizing domains in the Acss2 carboxy terminus. One region is homologous to a putative degron identified in an unbiased screen with protein fragments encoded by yeast genomic DNA[15]. The candidate degron region in Acss2 is largely disordered in structural determinations of both prokaryotic and yeast homologs[14, 17, 18]. The second region, the conserved basic domain, also has been postulated to function as an NLS and may exist as a partial alpha helical structure with a disordered domain. Both regions are inactive and likely masked in the latent state, but it is possible that these elements may be partially exposed while bound to substrates or products and performing its enzymatic function. The carboxy terminus of ACS, the prokaryotic homolog of Acss2, rotates following the first step of acetyl CoA generation, adenylation, and prior to the second and final step of the acetyl CoA generation, thioester formation[14, 18]. Mutation of a lysine residue in the conserved basic domain of ACS results in an inactive enzyme that is unable to proceed past adenylation and has impaired conformational fluidity[18].

Deletion of the initial amino terminus of the hinge region up to the degron likely exposes one or more destabilizing elements, resulting in marked instability of WT or ED fusion protein. Further amino truncations to the alpha 18 domain partially deletes the degron and results in a reversal of protein stability for WT versus ED fusion proteins, although ED fusion proteins retaining just eighteen amino acid residues comprising the ED ABC domain are considerably less stable than the parental moxGFP fusion protein. In addition, the increased protein levels of the moxGFP:ED ABC domain fusion protein when sequences carboxy to the ABC domain are removed suggests that this region may be involved in masking the ABC domain and thereby preventing it from destabilizing the Acss2 protein. Whether additional protein destabilization and masking domains are present in the hinge region of Acss2 remains to be determined.

Why is it important that Acss2 has multiple protein instability domains in its carboxy hinge region? The hinge region of Acss2 contains several features that are required for its enzymatic function[12, 14, 18]. In addition, the basic-rich region in Acss2 hinge region that we refer to as the ABC domain is involved in modulation of Acss2 subcellular localization and may therefore be essential for its role in nuclear signal transduction[5, 7, 8]. Unmasking this region in a controlled manner may expose the conserved basic domain in WT Acss2 protein and allow it to transit from the cytosol to nucleus. However, if a malformed and inactive Acss2 protein is unable to perform its primary function of acetyl CoA generation, its presence would not be desirable in either the cytosol or nucleus, particularly if they could still bind substrate or cofactors. In this case, clearance of any malfunctioning Acss2 proteins would benefit the cell.

Acss2 may contain multiple protein destabilization elements to ensure that mutations in one protein destabilization element that eliminate or reduce its protein instability capacity, as may be the case for the ED mutation, does not lead to retention of a malfunctioning protein. Detailed structural studies of WT and ED Acss2 protein may provide insights into how the ED mutation results in destabilization of Acss2 protein. However, there may be considerable technical hurdles preventing such an assessment as no structures of mammalian Acss2 have been published to date to the best of our knowledge. Molecular modeling of the isolated WT and ED Acss2 proteins suggests an overt conformational change in the ED Acss2 ABC domain, which is predicted to be localized on the surface of Acss2 (S1 Fig). Whether the ED mutation also results in more subtle conformational changes that activate the degron in the full-length Acss2 protein, which is also localized on the surface, is not known.

While the structural consequences of the ED mutation remain speculative, the results herein nevertheless may have important relevance for human genetic studies. The conserved basic domain in Acss2 is the most highly conserved stretch in Acss2 homologs from all organisms. Conformational changes in the Acss2 protein resulting from genetic or acquired lesions may unmask one or more protein destabilization domains and subsequently lead to a reduction in Acss2 protein levels. A systematic evaluation of the effect of substitutions in this domain may provide valuable insight into both structure-function aspects for Acss2 as well as potential insights into pathogenesis for humans exhibiting polymorphisms in this region.

## Materials and methods

### Bioinformatics

#### Homology comparisons

The Homo sapien/Human (NCBI reference sequence NP_061147.1), Mus musculus/House mouse (NCBI reference sequence NP_062785.2), Oryctolagus cuniculus/Rabbit (NCBI reference sequence XP_002710838.1), Ochotona princeps/American pika (NCBI reference sequence XP_004585804.1), Equus caballus/Horse (NCBI reference sequence XP_001501391.1), Rattus norvegicus/Norway rat (NCBI reference sequence NP_001101263.1), Bos taurus/Cow (NCBI reference sequence NP_001098809.1), Sus scrofa/Pig (NCBI reference sequence NP_001137167.1), and Ovis aries/Sheep (NCBI reference sequence XP_004014562.1) Acss2 proteins as well as the *Saccharomyces cerevisiae* Acss2 homolog Acs2p protein (NCBI reference sequence AAB35143.1) and the Acss2 homolog *Halobacterium salinarum* ACS protein (NCBI reference sequence AAG19414.1) were identified manually from the NCBI database and used in the indicated alignments. Sequences in the candidate basic-rich nuclear localization signal (NLS) residing in the Acss2 carboxy terminus were aligned and compared using Clustal Omega.

#### Molecular modeling

The protein sequences of the WT and ED mAcss2 proteins were used for structural modeling predictions with the use of a web-based integrated service, SWISS-MODEL[19]. Default parameters were used in modeling predictions with the SWISS-MODEL server homology modeling pipeline in conjunction with the repository comprising the SWISS-MODEL template library. The template with highest predicted correlation based upon the Global Model Quality Estimation (GMQE) and QMEAN Z-score was used for comparison. GMQE (Global Model Quality Estimation) is a quality estimation combining properties from target–template alignment and template search methods. The GMQE score (0 to 1) reflects the expected accuracy of a specific model with higher numbers indicating greater reliability. QMEAN is a composite estimator based on different geometrical properties that provides global as well as local absolute quality estimates of a single model. The QMEAN Z-score estimates how the "degree of nativeness" of global structural features observed in a model compares to expected experimental structures of similar size. QMEAN Z-scores close to zero indicate models with good agreement whereas QMEAN Z-scores of -4.0 or below indicate models with low quality. The colors in the surface models are based on QMEAN gradient scoring with blue and red representing well and poorly modeled regions, respectively. The SWISS-MODEL pipeline identified prokaryotic Acss2 homologs as template models for WT and ED mAcss2 protein with the crystal structure of acetyl-coenzyme A synthetase containing an R194A mutation as the highest match (SMTL ID: 2p2m.1)[18] in both cases. The GMQE score for WT and ED mAcss2 protein models was 0.76 with a QMEAN Z-score of -1.20 and -1.17, respectively.

### Animal studies

#### Mutant mouse generation

The mouse Acss2 gene was targeted for CRISPR/Cas9 by the University of Texas Southwestern Transgenic Core using a guide RNA (gRNA) designed and synthesized by the Genome Engineering Center at Washington University in St. Louis. The sequence of the gRNA targeting the mouse Acss2 gene (NCBI genomic sequence NC_000068.7, Reference GRCm38.p6 C57BL/6J) is as follows: GGTCATTCTGAGCAATCTTCcgg. The gRNA was synthesized using a T7 promoter-containing plasmid (Cat. # 43860, AddGene, Watertown, MA) and an RNA synthesis kit (Cat. # E2040S, HiScribe T7 High Yield RNA Synthesis Kit, New England Biolabs, Ipswich, MA). Cas9 RNA was synthesized using a T7 promoter-containing plasmid encoding mammalian optimized wild-type Cas9 protein (Cat. # 43945, AddGene, Watertown, MA) in conjunction with an RNA synthesis kit (Cat. # AM1345, mMESSAGE mMACHINE T7 Ultra Kit, Life Technologies, Carlsbad, CA). A repair oligo (IDT) designed to knock-in the ED mutation and a restriction site for screening (Xho I) had the following sequence: 5’-GAGGTGTGTGGGTTACCAGGGAAAATCATGAGGCGAGTTCTCGAGGATATTGCTCAGAATGACCATGACCTGGGGGATACATCTACGGTG-3’. The Acss2 gRNA, Cas9 mRNA, and repair oligo were co-injected into fertilized oocytes from C57BL/6N female mice to facilitate knock-in via homologous recombination. Founder mice were bred with C57BL/6J mice and progeny screened for the correct mutation by restriction fragment length polymorphism study and sequencing. After back-crossing correct founder mice for several generations with C57BL/6J mice, heterozygous mating pairs generated homozygous ED mice and WT litter-mates. All mice were maintained in standard bedding with microisolator caging under germ-free conditions at the animal facilities located at the University of Texas Southwestern, were fed and watered *ad lib* throughout the study, and were monitored for general health with sentinel mice. All mice used in experiments were generated from breeding colonies established in animal facilities located at the University of Texas Southwestern. All animal experiments were approved by the University of Texas Southwestern Institutional Animal Care and Use Committee (APN 2016–101616).

#### Anemia studies

Young adult mice received serial intra-peritoneal phenylhydrazine (PHZ; Cat. # 114715, Sigma, St. Louis, MO) injections to induce an acute hemolytic anemia and were analyzed as we have described previously in detail[2]. Briefly, PHZ was administered subcutaneously (intra-scapular) to isoflurane-anesthetized mice (1–2% using a vaporizer) at a dose of 40–80 μg PHZ/kg body weight using a 10 mg/ml PHZ (dissolved in sterile water) working stock, which typically involved injection of two 80–160 μL doses for a 20 gm mouse spaced ~24 hr apart. For the first four days following the initial PHZ injection, bedding and cages were treated as hazardous waste and disposed of or cleaned accordingly. Four days after the initial PHZ injection, hematocrits were measured in duplicates with blood obtained from retro-orbital eyebleeds. Mice with hematocrits outside the target experimental range (less than 19% or greater than 27%) were euthanatized. To assess recovery from acute anemia, blood was obtained one week prior to PHZ injection (baseline), four days following PHZ injection (nadir phase), and eight days following PHZ injection (recovery phase) for hematocrit determinations (n = 7 per genotype). For molecular and serum erythropoietin (Epo) studies, mice were euthanatized four days after PHZ injection for serum and tissue collection (n = 8 per genotype).

Moderate anemia induced by PHZ (19–27% versus ~45% hct for normal mice) generally produces no significant effects except for reduced maximal exertional capacity. Mice were observed daily following PHZ injection until euthanasia for respiratory effort, weight loss, grooming, porphyrin tears, spontaneous movement, gait or other motor abnormalities. Clinical signs that warranted euthanasia included if beginning four days after intervention there was weight loss >30% from initial weight, poor grooming or porphyrin tears, labored breathing at rest, reduced spontaneous movement, or severe gait or anatomical abnormalities that preclude adequate food and water intake. Euthanasia was performed in accordance with the University of Texas Southwestern Institutional Animal Care and Use Committee Euthanasia of Mice and Rats policy and the 2013 American Veterinary Medicine Association Guidelines on Euthanasia. Euthanasia was performed by inhalational anesthetic overdose of isoflurane with a secondary assurance method (cervical dislocation, decapitation, bilateral thoracotomy, exsanguination) or by carbon dioxide asphyxiation with a physical secondary assurance method (cervical dislocation, bilateral thoracotomy, exsanguination, organ removal). All animal experiments were approved by the University of Texas Southwestern Institutional Animal Care and Use Committee (APN 2016–101616).

#### Mouse embryonic fibroblast studies

Mouse embryonic fibroblasts (MEF) were isolated from WT and ED Acss2 littermate embryos derived from matings of heterozygous ED Acss2 mice as previously described[13]. Briefly, the uterus of an isoflurane-anesthetized pregnant female was removed 14 days post-coitus and rinsed in 1x PBS, and then the female was euthanized. The uterus was then submerged in 70% ethanol briefly and transferred to sterile 1x PBS. Individual embryos were removed from the uterus and separated from the placenta and yolk sacs. The head was removed and reserved for genotyping, and the legs, gut, and visceral organs were excised and discarded. The remaining tissue was minced with a sterile razor blade and transferred to a 15 ml conical tube. One ml of 0.25% trypsin-EDTA (Cat. # 25-053-CI, Corning, Manassas, VA) was added and incubated at 37°C for 10 min, with brief vortexing at 5 and 10 min. Two ml of DMEM containing 20% fetal bovine serum (FBS; Cat. # S11150, Atlanta Biologicals, Lawrenceville, GA) was added to inactivate trypsin, followed by centrifugation at 500g for 5 min. The supernatant was removed and the cell pellet was resuspended in 3 ml DMEM-20% FBS with 1x penicillin/streptomycin (P/S; Cat. # 30-002-CI, Corning, Manassas, VA), then passed through a 100 μm cell strainer into individual wells of a 6-well plate. After the cells attached approximately 6 hr later, media was replaced with cell incubation media, DMEM-10% FBS-1x P/S. The mouse head tissue was digested overnight in lysis buffer (50 mM Tris-pH 8.0, 10 mM EDTA, 100 mM NaCl, 0.1% sodium dodecyl sulfate, 300 μg/mL proteinase K) and genotyped the next day. MEF from homozygous WT or ED Acss2 embryos were expanded and frozen.

#### Molecular and serum studies

Mouse kidney and liver mRNA was prepared from individual organs (n = 8 mice per group). Mouse erythropoietin (Epo) and Acss2 mRNA levels for each organ were measured in triplicate by real-time PCR analysis as we have previously described in detail[2]. MEF were grown in 12-well plates as triplicate biological samples. MEF RNA was prepared from pooled wells of freshly confluent cells using a GenElute Mammalian Total RNA Miniprep kit (Cat. # RTN350, Sigma, St. Louis, MO). RNA was reverse-transcribed to cDNA using a High-Capacity cDNA Reverse Transcription Kit (Cat. # 4368813, Applied Biosystems, Foster City, CA). Mouse Acss2 mRNA levels for each MEF pool (n = 3 mice per group) were measured in triplicate for real-time PCR analysis. Mouse Acss2 mRNA levels in kidney, liver and MEF were measured by real-time PCR analysis using the following primer set: 5’-AAACACGCTCAGGGAAAATCA-3’ (forward), 5’-ACCGTAGATGTATCCCCCAGG-3’ (reverse). Mouse serum Epo protein was measured using a mouse erythropoietin Quantikine ELISA assay (Cat. # MEP00B, R&D Systems, Minneapolis, MN) for samples collected from WT and ED adult mice as we have previously described in detail[2].

Immunohistochemistry Studies: Kidney and liver samples from WT and ED adult mice were formalin-fixed, paraffin-embedded, and used for Acss2 immunohistochemical analysis as we previously described in detail[2].

#### Immunoblot studies

Protein samples prepared from liver, kidney, brain, heart, lung, and spleen samples (10 μg) as well as from MEF using CytoBuster protein extraction reagent (Cat. # 71009, Novagen, Gibbstown, NJ) with 1× protease inhibitor cocktail (Cat. #P8340, Sigma, St. Louis, MO). Extracts were electrophoresed and subjected to immunoblot analysis as previously described[2] with primary antibodies for the following antigens: human Acss2 (1:1,000 dilution; Cat. # 3658, Cell Signaling Technology, Danvers, MA), α-tubulin (1:3,000–1:5,000 dilution; Cat. # T9026, Sigma, St. Louis, MO).

#### Immunofluorescent microscopy studies with mouse embryonic fibroblasts

Acss2 ED and WT littermate MEF were plated onto chamber slides, incubated for 24 hr, fixed with ice cold methanol for 15 min at -20°C, then rinsed with 1x PBS. Cells were permeabilized with 0.5% Triton X-100 in 1x PBS for 10 min at room temperature, then rinsed once with 1x PBS. Slides were blocked for 30 min at room temperature in 5% normal goat serum, 1% fish skin gelatin (Cat. # G7765, Sigma, St. Louis, MO), 1% BSA (Cat. # A3059, Sigma, St. Louis, MO) in 1x PBS, then incubated in anti-Acss2 antibody (Cat. # 3658, Cell Signaling Technology, Danvers, MA) diluted 1:400 in blocking solution for 2 hr at room temperature. Slides were washed 3 times in 1x PBS, once in 1x PBS + 0.1% Triton X-100 for 1 min each wash, followed by a 1 hr room temperature incubation with Alexa 555 goat anti-rabbit secondary antibody (1:400 dilution; Cat. # 4413, Cell Signaling Technology, Danvers, MA). Slides were washed 3 times for 1 min in 1x PBS, then mounted with Vectashield containing DAPI (Cat. # H1200NB, Vector Labs, Burlingame, CA). Fluorescent images were obtained with 20X magnification using a Zeiss LSM 880 on an AxioObserver Z1 stand with Zen 2.3 Pro software (University of Texas Southwestern O’Brien Kidney Center Core).

### Stable cell culture studies

#### Cell culture maintenance

We maintained HeLa cells (Cat. # CCL-2, ATCC, Manassas, VA) and HEK293T cells (Cat. # CRL-3216, ATCC, Manassas, VA) in complete DMEM media (Cat. # SH30243.FS, GE Life Sciences, Pittsburg, PA), 10% fetal bovine serum (FBS; Cat. # S11150, Atlanta Biologicals, Lawrenceville, GA) with penicillin (100 U mL^−1^)/streptomycin (100 μg mL^−1^) (P/S; Cat. # 30-002-CI, Corning, Manassas, VA) in a 5% CO_2_, 95% air incubator. For passaging cells, we added 1 ml 0.25% Trypsin (Cat. # 25-053-CI, Corning, Manassas, VA) to a freshly confluent 10 cm plate and incubated at 37°C until dislodged. Trypsinized cells from one 10 cm plate were added to complete media (1:6 ratio) and re-plated. We used HeLa cells (unselected or selected) from between 15 and 25 passages, and HEK293 cells from between 3 and 8 passages, after recovery from liquid nitrogen stocks in all experiments.

#### Generation of moxGFP:Acss2 fusion protein-expressing lentivirus

We used derivatives of the lentiviral expression plasmid pLenti6/V5-GW/lacZ (Invitrogen) to generate lentiviruses expressing moxGFP:Acss2 fusion proteins. We first replaced the sequence encoding lacZ in pLenti6/V5-GW/lacZ with sequence encoding moxGFP (Cat. # 68070, Addgene, Watertown, MA) by standard molecular biological techniques. The carboxy terminal region from mouse Acss2 was then fused in-frame with the carboxy terminus of moxGFP. The amino truncated fusion constructs begin at the hinge region (amino acids 568 through 701), E1 region (amino acids 591 through 701), E2 region (amino acids 606 through 701), alpha 18 region (amino acids 619 through 701), or A9 region (amino acids 639 through 701). The isolated domain constructs include the degron homologous region (amino acids 632 through 653) and the candidate nuclear localization signal (NLS) domain (amino acids 653 through 671). All constructs except for the isolated degron region were made in two variants with either WT or ED encoding sequences.

We used the LTV packaging plasmids psPAX2 (Cat. # 12260, Addgene, Watertown, MA) and pMD2.G (Cat. # 12559, Addgene, Watertown, MA) in conjunction with the lentiviral expression plasmids to generate lentiviruses. We generated concentrated lentiviral stocks from a 10 cm plate of HEK293T cells co-transfected with 7 μg of psPAX2, 2 μg of pMD2.G, and 7 μg of the expression plasmid using polyethylenimine (PEI; Cat. #23966–1, Polysciences, Warrington, PA) as a transfection agent. Viral particles were pelleted by spinning at 24,000 rpm for 2 hours at 4°C, or by spinning at 1,500xg for 1 hour at room temperature following incubation in Lenti-X Concentrator (Cat. #631232, Takara, Mountain View, CA). Virus was aliquoted and stored in -80°C freezer until use.

#### Generation of moxGFP:Acss2 fusion protein-expressing stable cell lines

HeLa cells were transduced with lentivirus for 24 hr as we previously described for HT1080 cells[8]. Selection was performed using complete media containing 5 μg/ml blasticidin S (Cat. # ant-bl-1, Invivogen, San Diego, CA) for 4 d until all control cells had died. Positive cells were further cultured in 2 μg/ml blasticidin S for 2 wk prior to use in experiments. We used freshly confluent cells without drug selection in final experiments.

#### Immunoblot studies of moxGFP:Acss2 fusion proteins

HeLa stable cell lines were grown on 6-well tissue culture plates for 48 hours. Protein samples prepared from HeLa cells (10 μg) using CytoBuster protein extraction reagent (Cat. # 71009, Novagen, Gibbstown, NJ) with 1× protease inhibitor cocktail (Cat. #P8340, Sigma, St. Louis, MO) were subjected to immunoblot analysis following SDS-PAGE separation at 110V on a 10% polyacrylamide gel in SDS buffer (Cat. # 1610772, Bio-Rad, Hercules, CA) and transferred for 3.5 hours onto PVDF membrane (Cat. # 88520, ThermoFisher, Waltham, MA) at 65V in transfer buffer (Cat. # 1610771, Bio-Rad, Hercules, CA). Membranes were blocked for one hour at room temperature in blocking buffer (3% non-fat dry milk in TBS-T (TBS + 0.1% Tween-20) prior to overnight incubation with anti-GFP antibody (1:1,000 dilution in blocking buffer; Cat. # A11121, Invitrogen, Carlsbad, CA). The following day, membranes were washed 5 times for 5 min in TBS-T, then incubated for 1 hour at room temperature in HRP-linked, goat anti-mouse secondary AB (1:4,000 dilution; Cat. # 7076, Cell Signaling Technology, Danvers, MA). Membranes were washed again 5 times for 5 min in TBS-T and incubated in BioRad Clarity detection reagent (Cat. # 1705061, Bio-Rad, Hercules, CA) for 5 minutes before exposing to X-ray film (Cat. # F-BX810, Phenix Research Products, Candler, NC). For the tubulin loading control, membranes were first stripped with Thermo Restore (Cat. # 21059, ThermoFisher, Waltham, MA) for 15 minutes, washed twice for 15 minutes in TBS-T, and blocked for 30 min at room temperature in blocking buffer. Membranes were then immunostained for α-tubulin (1:5,000 dilution in blocking buffer; Cat. # T9026, Sigma, St. Louis, MO) as described for GFP. The secondary antibody was used at a 1:80,000 dilution.

### Statistical analyses

Where indicated, data were presented as mean with standard deviation (SD) or standard error of the mean (SEM). Statistical analyses were performed using Prism (Graphpad Software, San Diego, CA). We assumed equal variances for experimental groups. We compared results obtained from the indicated experimental groups by unpaired Student’s t-Test with Welch’s correction for groups of equal sample size. One-tailed or two-tailed analyses were performed as indicated with one-tailed analyses restricted to hematocrit, Epo mRNA and serum protein assessments based upon previous results with Acss2 null mice. All P values considered significant are less than or equal to 0.05 (*) or 0.10 (**) for the stated comparisons.

## Supporting information

S1 Table Source data used in generation of chart figures. The source data for the chart data presented in Figs 2A, 2B, 2C, 3C, 4C, and 6C are presented in separate worksheets within an Excel file.
(PDF)

S1 Fig Molecular modeling predictions of WT and ED mouse Acss2 proteins. (A) The amino acid sequences of WT (NCBI Reference Sequence NP_062785.2) and ED mouse Acss2 (mAcss2) protein was used in SWISS-MODEL for modeling predictions. Surface representations of WT and ED mAcss2 proteins are shown. The location of the parental amino acid residues (RK) in WT mAcss2 is indicated by a blue arrow and the substitution amino acid residues (ED) in ED mAcss2 is indicated by a red arrow. Loss of an electrostatic bridge is seen in ED mAcss2 protein compared with WT mAcss2 protein. (B) Tube representations of WT and ED mAcss2 proteins with parental residues in WT mAcss2 protein (RK) and substitution residues in ED mAcss2 protein (ED) indicated as above and also by ball and stick figures.
(PDF)
