# Supplementary material for: A substitution mutation in a conserved domain of mammalian acetate-dependent acetyl CoA synthetase 2 results in destabilized protein and impaired HIF-2 signaling
Source: PLoS One. 2019 Nov 14;14(11):e0225105. doi: 10.1371/journal.pone.0225105 (PMC6855420; doi:10.1371/journal.pone.0225105)

									53.5 mg/kg PHZ	53.5 mg/kg PHZ	day 0	day 0	day 0	day 0	day 0	day 4	day 4	day 4	day 4	day 4	day 8	day 8	day 8	day 8	day 8
Harvest Group	Mouse Number	Gender	Genotype	Treatment	DOB	Baseline Date	Treatment Dates	Harvest Date	1st Inj (μl)	2nd Inj (μl)	Baseline Wt (g)	Baseline Eye HCT/RBC Length (mm)	Baseline Eye HCT/Total Length (mm)	Baseline Eye HCT	Baseline Average HCT	Body Wt (g)	Eye HCT/RBC Length (mm)	Eye HCT/Total Length (mm)	Day 4 Eye HCT	Day 4 Average HCT	Body Wt (g)	Eye HCT/RBC Length (mm)	Eye HCT/Total Length (mm)	Eye HCT %	Average HCT
SC-2	28234	M	WT	PHZ time-course	10.06.15	12.09.15	12.14.15/ 12.15.15	12.18.15/ 12.22.15	92.1	92.1	24.6	12.0	26.0	46.2	47.3	22.1	5.0	18.0	27.8	27.5	26.1	10.0	20.0	50.0	50.0
												15.0	31.0	48.4			6.0	22.0	27.3			10.0	20.0	50.0	
SC-2	28244	M	WT	PHZ time-course	10.06.15	12.09.15	12.14.15/ 12.15.15	12.18.15/ 12.22.15	84.6	84.6	22.6	12.0	25.0	48.0	48.0	22.0	6.0	22.0	27.3	27.5	24.3	10.0	22.0	45.5	45.5
												12.0	25.0	48.0			5.0	18.0	27.8			10.0	22.0	45.5	
SC-2	28245	M	WT	PHZ time-course	10.06.15	12.09.15	12.14.15/ 12.15.15	12.18.15/ 12.22.15	85.8	85.8	22.9	12.0	26.0	46.2	46.2	20.5	7.0	26.0	26.9	26.0	23.2	9.0	19.0	47.4	47.6
												12.0	26.0	46.2			6.0	24.0	25.0			11.0	23.0	47.8	
SC-2	28263	M	WT	PHZ time-course	10.12.15	12.09.15	12.14.15/ 12.15.15	12.18.15/ 12.22.15	76.4	76.4	20.4	10.0	21.0	47.6	48.8	17.3	4.0	20.0	20.0	20.5	19.6	9.0	21.0	42.9	43.9
												10.0	20.0	50.0			4.0	19.0	21.1			9.0	20.0	45.0	
SC-2	28269	M	WT	PHZ time-course	10.12.15	12.09.15	12.14.15/ 12.15.15	12.18.15/ 12.22.15	77.9	77.9	20.8	12.0	24.0	50.0	48.7	18.5	5.0	20.0	25.0	24.4	21.4	11.0	23.0	47.8	47.8
												9.0	19.0	47.4			5.0	21.0	23.8			11.0	23.0	47.8	
SC-5	28334	F	WT	PHZ time-course	11.14.15	01.20.16	01.25.16/ 01.26.16	01.29.16/ 02.02.16	94.5	94.5	18.9	10.0	20.0	50.0	48.9	16.1	5.0	24.0	20.8	21.3	19.9	9.0	19.0	47.4	46.2
												11.0	23.0	47.8			5.0	23.0	21.7			9.0	20.0	45.0	
SC-5	28346	F	WT	PHZ time-course	11.24.15	01.20.16	01.25.16/ 01.26.16	01.29.16/ 02.02.16	100.5	100.5	20.1	13.0	26.0	50.0	47.9	17.1	4.0	16.0	25.0	26.3	20.3	10.0	19.0	52.6	52.6
												11.0	24.0	45.8			8.0	29.0	27.6			10.0	19.0	52.6	
														mean	48.0				mean	24.8				mean	47.7
														n	7				n	7				n	7
														STD	1.0				STD	2.9				STD	2.9
														SEM	0.4				SEM	1.1				SEM	1.1
SC-1	28166	M	CYT HOM	PHZ time-course	09.20.15	12.02.15	12.07.15/ 12.08.15	12.11.15/ 12.15.15	99.2	99.2	26.5	13.0	26.0	50.0	48.7	23.9	5.0	20.0	25.0	25.0	27.3	10.0	23.0	43.5	43.5
												9.0	19.0	47.4			5.0	20.0	25.0			10.0	23.0	43.5	
SC-1	28168	M	CYT HOM	PHZ time-course	09.20.15	12.02.15	12.07.15/ 12.08.15	12.11.15/ 12.15.15	103.7	103.7	27.7	11.0	23.0	47.8	47.8	24.8	5.0	19.0	26.3	26.3	25.6	7.0	18.0	38.9	38.5
												11.0	23.0	47.8			5.0	19.0	26.3			8.0	21.0	38.1	
SC-1	28177	M	CYT HOM	PHZ time-course	09.21.15	12.02.15	12.07.15/ 12.08.15	12.11.15/ 12.15.15	88.8	88.8	23.7	10.0	21.0	47.6	47.6	21.2	4.0	19.0	21.1	23.0	24.3	10.0	22.0	45.5	45.5
												10.0	21.0	47.6			6.0	24.0	25.0			10.0	22.0	45.5	
SC-2	28261	M	CYT HOM	PHZ time-course	10.10.15	12.09.15	12.14.15/ 12.15.15	12.18.15/ 12.22.15	91.0	91.0	24.3	15.0	30.0	50.0	48.9	21.6	5.0	20.0	25.0	25.0	24.8	10.0	20.0	50.0	50.0
												11.0	23.0	47.8			6.0	24.0	25.0			10.0	20.0	50.0	
SC-5	28339	M	CYT HOM	PHZ time-course	11.14.15	01.20.16	01.25.16/ 01.26.16	01.29.16/ 02.02.16	120.0	120.0	24.0	8.0	17.0	47.1	47.4	19.9	3.0	16.0	18.8	19.4	19.0	4.0	12.1	33.1	30.0
												11.0	23.0	47.8			4.0	20.0	20.0			3.0	11.1	27.0	
SC-2	28257	F	CYT HOM	PHZ time-course	10.10.15	12.09.15	12.14.15/ 12.15.15	12.18.15/ 12.22.15	72.7	72.7	19.4	10.0	20.0	50.0	48.7	16.3	4.0	18.0	22.2	22.2	20.0	9.0	19.0	47.4	47.4
												9.0	19.0	47.4			4.0	18.0	22.2			9.0	19.0	47.4	
SC-2	28258	F	CYT HOM	PHZ time-course	10.10.15	12.09.15	12.14.15/ 12.15.15	12.18.15/ 12.22.15	71.9	71.9	19.2	10.0	20.0	50.0	50.0	17.0	5.0	20.0	25.0	25.0	20.1	10.0	20.0	50.0	48.7
												10.0	20.0	50.0			5.0	20.0	25.0			9.0	19.0	47.4	
														mean	48.5				mean	23.7				mean	43.4
														n	7				n	7				n	7
														STD	0.9				STD	2.4				STD	7.0
														SEM	0.3				SEM	0.9				SEM	2.6

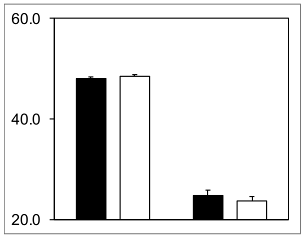

		day 0	day 4	n
Mean	WT	48.0	24.8	7
	CYT HOM	48.5	23.7	7
SEM	WT	0.4	1.1	7
	CYT HOM	0.3	0.9	7

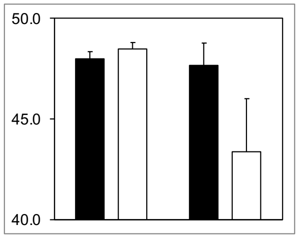

		day 0	day 8	n
Mean	WT	48.0	47.7	7
	CYT HOM	48.5	43.4	7
SEM	WT	0.4	1.1	7
	CYT HOM	0.3	2.6	7

Unpaired t test	
P value	0.0807
P value summary	ns
Significantly different (P < 0.1)?	Yes
One- or two-tailed P value?	One-tailed
t, df	t=1.492, df=12

Final Group	Harvest Group	Mouse Number	Gender	Genotype	Treatment	DOB	Baseline Date	Baseline Weight (g)	Baseline Eye Hct/RBC Length	Baseline Eye Hct/Total Length	Baseline Eye Hct	Average Baseline Eye Hct/Select	Harvest Date	Harvest Weight (g)	Harvest Eye Hct/RBC Length	Harvest Eye Hct/Total Length	Harvest Eye Hct	Average Harvest Eye Hct/Select	Kidney RNA (ng/uL)	Kidney RNA (uL/0.5 ug)	Liver RNA (ng/uL)	Liver RNA (uL/0.5 ug)
																			(FastPrep)		(BioRad)	
WT Control	1	27221	M	WT	Control (harvest Day 4)	02.22.15	04.20.15	22.7	8	16	50.0	50.0	04.28.15	20.4	8	17	47.1	50.0	272.7	1.8	662.2	0.8
									9	18	50.0				9	17	52.9					
WT Control	2	27244	M	WT	Control (harvest Day 4)	03.01.15	04.20.15	25.5	8	17	47.1	45.0	04.29.15	23.1	7	14	50.0	50.0	470.1	1.1	444.2	1.1
									9	21	42.9				8	16	50.0					
WT Control	6	27299	M	WT	Control (harvest Day 4)	03.22.15	05.12.15	24.1	6	12	50.0	48.5	05.19.15	21.6	12	24	50.0	50.0	150.5	3.3	639.3	0.8
									8	17	47.1				9	18	50.0					
WT Control	18	27860	M	WT	Control (harvest Day 4)	07.14.15	09.13.15	23.4	10	20	50.0	48.7	09.17.15	19.9	7	16	43.8	45.4	210	2.4	560.7	0.9
									9	19	47.4				8	17	47.1					
WT Control	3	27238	F	WT	Control (harvest Day 4)	03.01.15	04.20.15	22.3	9	18	50.0	50.0	04.30.15	20.2	8	16	50.0	50.0	395.7	1.3	742.4	0.7
									9	18	50.0				8	16	50.0					
WT Control	4	27250	F	WT	Control (harvest Day 4)	03.03.15	04.20.15	17.5	8	17	47.1	47.1	05.01.15	16.0	10	19	52.6	51.3	286.5	1.7	641.0	0.8
									8	17	47.1				10	20	50.0					
WT Control	6	27303	F	WT	Control (harvest Day 4)	03.22.15	05.12.15	16.7	9	18	50.0	50.0	05.19.15	14.4	9	18	50.0	48.7	158.0	3.2	685.2	0.7
									9	18	50.0				9	19	47.4					
WT Control	18	27881	F	WT	Control (harvest Day 4)	07.13.15	09.13.15	19.6	8	17	47.1	47.4	09.17.15	16.8	8	18	44.4	44.4	139.3	3.6	800.1	0.6
									11	23	47.8				8	18	44.4					
											mean	48.33					mean	48.73				
											n	8					n	8				
											STD	1.79					STD	2.47				
											SEM	0.63					SEM	0.87				
ED Control	14	27769	M	CYT HOM	Control (harvest Day 4)	06.19.15	08.14.15	25.3	9	19	47.4	48.7	08.24.15	22.4	15	30	50.0	50.0	227.8	2.2	569.9	0.9
									8	16	50.0				10	20	50.0					
ED Control	14	27770	M	CYT HOM	Control (harvest Day 4)	06.19.15	08.14.15	25.5	9	18	50.0	50.0	08.24.15	23.5	11	21	52.4	51.2	237.4	2.1	584.9	0.9
									9	18	50.0				9	18	50.0					
ED Control	14	27771	M	CYT HOM	Control (harvest Day 4)	06.19.15	08.14.15	23.3	10	20	50.0	50.0	08.24.15	20.8	9	18	50.0	48.5	288.1	1.7	632.0	0.8
									10	20	50.0				8	17	47.1					
ED Control	21	27887	M	CYT HOM	Control (harvest Day 4)	07.14.15	09.18.15	24.0	10	23	43.5	43.7	09.23.15	20.9	9	19	47.4	45.9	208.3	2.4	435.5	1.1
									11	25	44.0				8	18	44.4					
ED Control	24	28045	F	CYT HOM	Control (harvest Day 4)	08.24.15	10.05.15	17.0	8	17	47.1	46.0	10.05.15	17.0	8	17	47.1	46.0	428.1	1.2	307.8	1.6
									9	20	45.0				9	20	45.0					
ED Control	23	27962	F	CYT HOM	Control (harvest Day 4)	07.28.15	09.30.15	16.6	11	23	47.8	50.2	09.30.15	16.6	11	23	47.8	50.2	213.07	2.3	394.5	1.3
									10	19	52.6				10	19	52.6					
ED Control	11	27368	F	CYT HOM	Control (harvest Day 4)	05.01.15	06.22.15	16.3	8	16	50.0	50.0	06.30.15	14.0	9	18	50.0	48.3	168.4	3.0	432.0	1.2
									8	16	50.0				7	15	46.7					
ED Control	19	27902	F	CYT HOM	Control (harvest Day 4)	07.14.15	09.13.15	17.2	7	15	46.7	46.9	09.18.15	15.9	9	21	50.0	48.3	205.4	2.4	294.9	1.7
									8	17	47.1				8	19	46.7					
											mean	48.19					mean	48.56				
											n	8					n	8				
											STD	2.40					STD	1.90				
											SEM	0.85					SEM	0.67				
WT PHZ	9	27325	M	WT	PHZ (harvest Day 4)	04.17.15	06.08.15	19.2	8	16	50.0	50.0	06.19.15	18.5	3	14	21.4	21.8	443.7	1.1	414.7	1.2
									8	16	50.0				4	18	22.2					
WT PHZ	15	27795	M	WT	PHZ (harvest Day 4)	06.24.15	08.27.15	23.6	10	20	50.0	51.1	08.31.15	20.5	3	16	18.8	21.1	457.3	1.1	303.8	1.6
									12	23	52.2				4	17	23.5					
WT PHZ	15	27798	M	WT	PHZ (harvest Day 4)	06.24.15	08.27.15	25.0	9	18	50.0	50.0	08.31.15	21.1	3	15	20.0	21.1	472.8	1.1	416.6	1.2
									10	20	50.0				4	18	22.2					
WT PHZ	16	27816	M	WT	PHZ (harvest Day 4)	07.04.15	08.30.15	24.2	9	18	50.0	48.5	09.03.15	21.4	4	17	23.5	22.3	557.4	0.9	355.2	1.4
									8	17	47.1				4	19	21.1					
WT PHZ	8	27313	F	WT	PHZ (harvest Day 4)	03.28.15	05.18.15	15.0	7	15	46.7	46.9	05.29.15	14.9	5	18	27.8	26.4	473.0	1.1	378.6	1.3
									8	17	47.1				4	16	25.0					
WT PHZ	16	27857	F	WT	PHZ (harvest Day 4)	07.06.15	08.30.15	16.5	8	16	50.0	48.5	09.03.15	13.7	4	20	20.0	20.5	366.6	1.4	331.3	1.5
									8	17	47.1				4	19	21.1					
WT PHZ	16	27819	F	WT	PHZ (harvest Day 4)	07.04.15	08.30.15	17.7	9	19	47.4	47.5	09.03.15	14.6	4	21	19.0	19.5	432.2	1.2	433.5	1.2
									10	21	47.6				5	25	20.0					
WT PHZ	20	27933	F	WT	PHZ (harvest Day 4)	07.21.15	09.18.15	17.8	11	23	47.8	46.6	09.22.15	12.8	4	18	22.2	21.1	406.7	1.2	186.5	2.7
									10	22	45.5				4	20	20.0					
											mean	48.6					mean	21.7				
											n	8					n	8				
											STD	1.6					STD	2.1				
											SEM	0.6					SEM	0.7				
ED PHZ	12	27706	M	CYT HOM	PHZ (harvest Day 4)	05.10.15	07.05.15	22.0	8	16	50.0	51.7	07.13.15	22.0	4	17	23.5	24.3	418.1	1.2	404.7	1.2
									8	15	53.3				5	20	25.0					
ED PHZ	16	27833	M	CYT HOM	PHZ (harvest Day 4)	07.06.15	08.30.15	23.6	10	20	50.0	48.3	09.03.15	22.0	5	20	25.0	25.0	436.4	1.1	278.9	1.8
									7	15	46.7				4	16	25.0					
ED PHZ	9	27332	M	CYT HOM	PHZ (harvest Day 4)	06.08.15	06.08.15	23.0	9	18	50.0	50.0	06.19.15	23.6	4	17	23.5	23.7	562.5	0.9	314.3	1.6
									9	18	50.0				5	21	23.8					
ED PHZ	16	27831	M	CYT HOM	PHZ (harvest Day 4)	07.06.15	08.30.15	23.1	9	18	50.0	50.0	09.03.15	20.8	4	17	23.5	22.9	472.8	1.1	495.7	1.0
									8	16	50.0				4	18	22.2					
ED PHZ	24	27961	F	CYT HOM	PHZ (harvest Day 4)	07.28.15	10.05.15	18.1	10	21	47.6	47.8	10.05.15	13.2	4	21	19.0	19.5	424.9	1.2	447.4	1.1
									12	25	48.0				4	20	20.0					
ED PHZ	18	27897	F	CYT HOM	PHZ (harvest Day 4)	07.12.15	09.13.15	20.7	9	20	45.0	47.5	09.17.15	18.9	5	20	25.0	25.0	427.2	1.2	232.6	2.1
									11	22	50.0				5	20	25.0					
ED PHZ	18	27896	F	CYT HOM	PHZ (harvest Day 4)	07.12.15	09.13.15	18.8	9	18	50.0	48.7	09.17.15	16.4	4	18	22.2	21.6	459.2	1.1	223.3	2.2
									9	19	47.4				4	19	21.1					
ED PHZ	19	27891	F	CYT HOM	PHZ (harvest Day 4)	07.14.15	09.13.15	16.8	7	15	46.7	46.9	09.18.15	14.7	5	20	25.0	24.5	323.9	1.5	374.0	1.3
									8	17	47.1				6	25	24.0					
											mean	48.9					mean	23.3				
											n	8					n	8				
											STD	1.6					STD	1.9				
											SEM	0.6					SEM	0.7				

	AVG Control (Day 0)	AVG PHZ Day 4	SEM Control (Day 0)	SEM PHZ Day 4
WT	48.6	21.7	0.6	0.7
CYT HOM	48.9	23.3	0.6	0.7
n	8	8	8	8

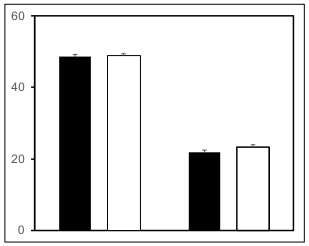

Treatment	Number	Acss2 Geno	Gender	mCyc Ct	mEpo Ct		dCt	dCT of normalizer	ddCt	quantity
Control (harvest Day 4)	27221	WT	M	25.57	Undetermined		Undetermined	19.69	Undetermined	Undetermined
Control (harvest Day 4)	27244	WT	M	24.20	Undetermined		Undetermined	19.69	Undetermined	Undetermined
Control (harvest Day 4)	27299	WT	M	22.29	Undetermined		Undetermined	19.69	Undetermined	Undetermined
Control (harvest Day 4)	27860	WT	M	23.36	41.14		17.77	19.69	-1.92	3.78
Control (harvest Day 4)	27238	WT	F	22.88	Undetermined		Undetermined	19.69	Undetermined	Undetermined
Control (harvest Day 4)	27250	WT	F	26.56	Undetermined		Undetermined	19.69	Undetermined	Undetermined
Control (harvest Day 4)	27303	WT	F	22.91	Undetermined		Undetermined	19.69	Undetermined	Undetermined
Control (harvest Day 4)	27881	WT	F	20.43	42.04		21.61	19.69	1.92	0.26
						mean	19.69			2.02
						n	2			2
						STD	2.71			2.48
						SEM	1.92			1.76
PHZ (harvest Day 4)	27325	WT	M	22.41	35.82		13.40	19.69	-6.29	78.12
PHZ (harvest Day 4)	27795	WT	M	23.49	34.08		10.59	19.69	-9.11	550.71
PHZ (harvest Day 4)	27798	WT	M	23.59	34.22		10.64	19.69	-9.05	531.62
PHZ (harvest Day 4)	27816	WT	M	24.14	36.13		11.99	19.69	-7.70	207.93
PHZ (harvest Day 4)	27313	WT	F	23.22	34.45		11.22	19.69	-8.47	354.44
PHZ (harvest Day 4)	27857	WT	F	25.08	35.67		10.59	19.69	-9.10	548.02
PHZ (harvest Day 4)	27819	WT	F	24.75	33.73		8.98	19.69	-10.71	1674.36
PHZ (harvest Day 4)	27933	WT	F	25.86	38.57		12.71	19.69	-6.98	126.53
						mean	11.27			574.27
						n	8			8
						STD	1.40			508.26
						SEM	0.49			179.70
PHZ (harvest Day 8)	28234	WT	M	20.09	41.32		21.23	19.69	1.54	0.34
PHZ (harvest Day 8)	28244	WT	M	20.87	40.35		19.48	19.69	-0.21	1.16
PHZ (harvest Day 8)	28245	WT	M	20.82	40.60		19.78	19.69	0.09	0.94
PHZ (harvest Day 8)	28263	WT	M	22.59	39.74		17.15	19.69	-2.55	5.84
PHZ (harvest Day 8)	28334	WT	F	20.17	40.26		20.09	19.69	0.40	0.76
PHZ (harvest Day 8)	28346	WT	F	21.01	40.92		19.91	19.69	0.22	0.86
PHZ (harvest Day 8)	28396	WT	F	20.08	37.45		17.37	19.69	-2.33	5.02
PHZ (harvest Day 8)	33550	WT	F	20.03	39.14		19.11	19.69	-0.58	1.50
						mean	19.26			2.13
						n	8			8
						STD	1.38			2.12
						SEM	0.49			0.75
Control (harvest Day 4)	27769	CYT HOM	M	23.00	Undetermined		Undetermined	19.69	Undetermined	Undetermined
Control (harvest Day 4)	27770	CYT HOM	M	23.51	Undetermined		Undetermined	19.69	Undetermined	Undetermined
Control (harvest Day 4)	27771	CYT HOM	M	22.93	Undetermined		Undetermined	19.69	Undetermined	Undetermined
Control (harvest Day 4)	27887	CYT HOM	M	22.72	41.15		18.42	19.69	-1.27	2.41
Control (harvest Day 4)	28045	CYT HOM	F	23.13	41.11		17.99	19.69	-1.71	3.26
Control (harvest Day 4)	27962	CYT HOM	F	22.94	Undetermined		Undetermined	19.69	Undetermined	Undetermined
Control (harvest Day 4)	27368	CYT HOM	F	23.98	Undetermined		Undetermined	19.69	Undetermined	Undetermined
Control (harvest Day 4)	27902	CYT HOM	F	23.64	Undetermined		Undetermined	19.69	Undetermined	Undetermined
						mean	18.20			2.84
						n	2			2
						STD	0.31			0.60
						SEM	0.22			0.42
PHZ (harvest Day 4)	27706	CYT HOM	M	23.12	39.04		15.92	19.69	-3.77	13.63
PHZ (harvest Day 4)	27833	CYT HOM	M	22.94	36.85		13.91	19.69	-5.78	54.95
PHZ (harvest Day 4)	27332	CYT HOM	M	22.72	36.17		13.45	19.69	-6.24	75.48
PHZ (harvest Day 4)	27831	CYT HOM	M	23.24	37.99		14.75	19.69	-4.94	30.73
PHZ (harvest Day 4)	27961	CYT HOM	F	24.80	36.21		11.41	19.69	-8.28	310.64
PHZ (harvest Day 4)	27897	CYT HOM	F	23.23	37.40		14.17	19.69	-5.52	46.03
PHZ (harvest Day 4)	27896	CYT HOM	F	23.41	35.90		12.49	19.69	-7.20	146.92
PHZ (harvest Day 4)	27891	CYT HOM	F	23.05	35.09		12.04	19.69	-7.65	201.16
						mean	13.52			109.94
						n	8			8
						STD	1.49			102.69
						SEM	0.53			36.31
PHZ (harvest Day 8)	28166	CYT HOM	M	20.12	40.49		20.37	19.69	0.68	0.63
PHZ (harvest Day 8)	28168	CYT HOM	M	20.70	37.33		16.63	19.69	-3.07	8.37
PHZ (harvest Day 8)	28177	CYT HOM	M	20.40	40.22		19.82	19.69	0.12	0.92
PHZ (harvest Day 8)	28261	CYT HOM	M	20.27	41.52		21.24	19.69	1.55	0.34
PHZ (harvest Day 8)	28257	CYT HOM	F	20.42	41.42		21.00	19.69	1.31	0.40
PHZ (harvest Day 8)	28258	CYT HOM	F	20.36	40.42		20.07	19.69	0.37	0.77
PHZ (harvest Day 8)	28386	CYT HOM	F	20.05	38.52		18.46	19.69	-1.23	2.34
PHZ (harvest Day 8)	33575	CYT HOM	F	19.78	39.75		19.97	19.69	0.28	0.82
						mean	19.69			1.83
						n	8			8
						STD	1.50			2.72
						SEM	0.53			0.96

	AVG		SEM	
	Control (Day 0)	PHZ Day 4	Control (Day 0)	PHZ Day 4
WT	2.02	574.27	1.76	179.70
CYT HOM	2.84	109.94	0.42	36.31
n	8	8	8	8

	AVG		SEM	
normalized	Control (Day 0)	PHZ Day 4	Control (Day 0)	PHZ Day 4
WT	1.00	284.07	0.87	88.89
CYT HOM	1.40	54.38	0.21	17.96
n	8	8	8	8

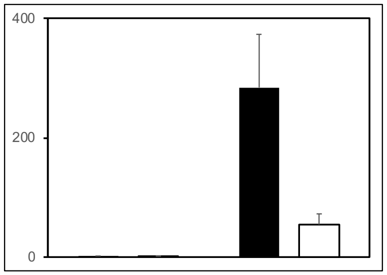

Treatment	Number	Acss2 Geno	Gender	mCyc Ct	mEpo Ct		dCt	dCT of normalizer	ddCt	quantity
Control (harvest Day 4)	27221	WT	M	21.61	Undetermined		Undetermined	21.03	Undetermined	Undetermined
Control (harvest Day 4)	27244	WT	M	21.17	Undetermined		Undetermined	21.03	Undetermined	Undetermined
Control (harvest Day 4)	27299	WT	M	20.82	Undetermined		Undetermined	21.03	Undetermined	Undetermined
Control (harvest Day 4)	27860	WT	M	21.61	Undetermined		Undetermined	21.03	Undetermined	Undetermined
Control (harvest Day 4)	27238	WT	F	22.01	Undetermined		Undetermined	21.03	Undetermined	Undetermined
Control (harvest Day 4)	27250	WT	F	21.41	Undetermined		Undetermined	21.03	Undetermined	Undetermined
Control (harvest Day 4)	27303	WT	F	21.42	Undetermined		Undetermined	21.03	Undetermined	Undetermined
Control (harvest Day 4)	27881	WT	F	21.19	Undetermined		Undetermined	21.03	Undetermined	Undetermined
						mean	#DIV/0!			#DIV/0!
						n	8			8
						STD	#DIV/0!			#DIV/0!
						SEM	#DIV/0!			#DIV/0!
PHZ (harvest Day 4)	27325	WT	M	21.25	41.88		20.63	21.03	-0.39	1.31
PHZ (harvest Day 4)	27795	WT	M	22.05	36.33		14.28	21.03	-6.75	107.70
PHZ (harvest Day 4)	27798	WT	M	22.02	36.33		14.31	21.03	-6.72	105.08
PHZ (harvest Day 4)	27816	WT	M	21.91	36.83		14.92	21.03	-6.11	68.95
PHZ (harvest Day 4)	27313	WT	F	22.03	36.04		14.01	21.03	-7.02	129.74
PHZ (harvest Day 4)	27857	WT	F	22.16	35.03		12.87	21.03	-8.16	285.93
PHZ (harvest Day 4)	27819	WT	F	22.57	35.03		12.46	21.03	-8.57	379.87
PHZ (harvest Day 4)	27933	WT	F	21.99	34.93		12.95	21.03	-8.08	270.47
						mean	14.55			168.63
						n	8			8
						STD	2.60			128.70
						SEM	0.92			45.50
PHZ (harvest Day 8)	28234	WT	M	21.61	Undetermined		Undetermined	21.03	Undetermined	Undetermined
PHZ (harvest Day 8)	28244	WT	M	21.17	Undetermined		Undetermined	21.03	Undetermined	Undetermined
PHZ (harvest Day 8)	28245	WT	M	20.82	Undetermined		Undetermined	21.03	Undetermined	Undetermined
PHZ (harvest Day 8)	28263	WT	M	21.61	Undetermined		Undetermined	21.03	Undetermined	Undetermined
PHZ (harvest Day 8)	28334	WT	F	22.01	Undetermined		Undetermined	21.03	Undetermined	Undetermined
PHZ (harvest Day 8)	28346	WT	F	21.41	Undetermined		Undetermined	21.03	Undetermined	Undetermined
PHZ (harvest Day 8)	28396	WT	F	21.42	42.45		21.03	21.03	0.00	1.00
PHZ (harvest Day 8)	33550	WT	F	21.19	Undetermined		Undetermined	21.03	Undetermined	Undetermined
						mean	21.03			1.00
						n	8			8
						STD	#DIV/0!			#DIV/0!
						SEM	#DIV/0!			#DIV/0!
Control (harvest Day 4)	27769	CYT HOM	M	20.55	Undetermined		Undetermined	21.03	Undetermined	Undetermined
Control (harvest Day 4)	27770	CYT HOM	M	20.56	Undetermined		Undetermined	21.03	Undetermined	Undetermined
Control (harvest Day 4)	27771	CYT HOM	M	21.18	Undetermined		Undetermined	21.03	Undetermined	Undetermined
Control (harvest Day 4)	27887	CYT HOM	M	21.67	Undetermined		Undetermined	21.03	Undetermined	Undetermined
Control (harvest Day 4)	28045	CYT HOM	F	20.48	Undetermined		Undetermined	21.03	Undetermined	Undetermined
Control (harvest Day 4)	27962	CYT HOM	F	21.03	Undetermined		Undetermined	21.03	Undetermined	Undetermined
Control (harvest Day 4)	27368	CYT HOM	F	21.36	Undetermined		Undetermined	21.03	Undetermined	Undetermined
Control (harvest Day 4)	27902	CYT HOM	F	21.25	Undetermined		Undetermined	21.03	Undetermined	Undetermined
						mean	#DIV/0!			#DIV/0!
						n	8			8
						STD	#DIV/0!			#DIV/0!
						SEM	#DIV/0!			#DIV/0!
PHZ (harvest Day 4)	27706	CYT HOM	M	21.82	40.95		19.13	21.03	-1.89	3.71
PHZ (harvest Day 4)	27833	CYT HOM	M	21.47	40.15		18.68	21.03	-2.35	5.10
PHZ (harvest Day 4)	27332	CYT HOM	M	21.76	39.82		18.06	21.03	-2.97	7.82
PHZ (harvest Day 4)	27831	CYT HOM	M	21.95	39.54		17.59	21.03	-3.44	10.83
PHZ (harvest Day 4)	27961	CYT HOM	F	21.05	37.35		16.30	21.03	-4.73	26.54
PHZ (harvest Day 4)	27897	CYT HOM	F	22.04	41.20		19.15	21.03	-1.88	3.67
PHZ (harvest Day 4)	27896	CYT HOM	F	23.07	36.52		13.45	21.03	-7.58	190.84
PHZ (harvest Day 4)	27891	CYT HOM	F	21.55	37.27		15.72	21.03	-5.31	39.55
						mean	17.26			36.01
						n	8			8
						STD	1.99			63.86
						SEM	0.70			22.58
PHZ (harvest Day 8)	28166	CYT HOM	M	20.55	Undetermined		Undetermined	21.03	Undetermined	Undetermined
PHZ (harvest Day 8)	28168	CYT HOM	M	20.56	40.86		20.30	21.03	-0.73	1.65
PHZ (harvest Day 8)	28177	CYT HOM	M	21.18	Undetermined		Undetermined	21.03	Undetermined	Undetermined
PHZ (harvest Day 8)	28261	CYT HOM	M	21.67	Undetermined		Undetermined	21.03	Undetermined	Undetermined
PHZ (harvest Day 8)	28257	CYT HOM	F	20.48	Undetermined		Undetermined	21.03	Undetermined	Undetermined
PHZ (harvest Day 8)	28258	CYT HOM	F	21.03	41.43		20.40	21.03	-0.63	1.55
PHZ (harvest Day 8)	28386	CYT HOM	F	21.36	Undetermined		Undetermined	21.03	Undetermined	Undetermined
PHZ (harvest Day 8)	33575	CYT HOM	F	21.25	Undetermined		Undetermined	21.03	Undetermined	Undetermined
						mean	20.35			1.60
						n	2			2
						STD	0.07			0.08
						SEM	0.05			0.05

	AVG		SEM	
	Control Day 4	PHZ Day 4	Control Day 4	PHZ Day 4
WT	0.00	168.63	0.00	45.50
CYT HOM	0.00	36.01	0.00	22.58
n	8	8	8	8

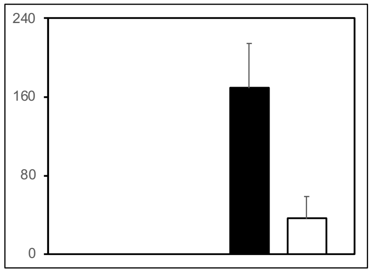

STANDARD CURVE - unadjusted

Original absorbance		Average	pg	Position	Name
0.460	0.459	0.460	3000	S1	standard 3000
0.282	0.315	0.299	1500	S2	standard 1500
0.187	0.193	0.190	750	S3	standard 750
0.152	0.152	0.152	375	S4	standard 375
0.126	0.124	0.125	188	S5	standard 188
0.128	0.115	0.122	94	S6	standard 94
0.115	0.109	0.112	47	S7	standard 47
0.107	0.122	0.115	0	S8	standard 0

STANDARD CURVE - adjusted

Adjusted absorbance		Average	pg	Position	Name
0.353	0.337	0.345	3000	S1	standard 3000
0.175	0.193	0.184	1500	S2	standard 1500
0.080	0.071	0.076	750	S3	standard 750
0.045	0.030	0.038	375	S4	standard 375
0.019	0.002	0.011	188	S5	standard 188
0.021	-0.007	0.007	94	S6	standard 94
0.008	-0.013	-0.003	47	S7	standard 47
0.000	0.000	0.000	0	S8	standard 0

	**AVG**		**SEM**	
	**PHZ Day 0**	**PHZ Day 4**	**PHZ Day 0**	**PHZ Day 4**
**WT**	80	52,192	41	12,624
**CYT HOM**	33	17,827	18	6,004
**n**	8	8	8	8

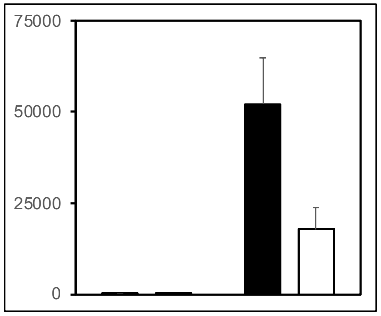

Group	Treatment	Number	Genotype	Gender	Abs 1st	Abs 2nd	Ave Abs	Epo (pg)	Corrected Epo	Adjusted Epo	Mean	SD	SEM	n
1	Control	27221	WT	M	0.007	0.019	0.013	164	328	328				
2	Control	27244	WT	M	0.006	0.002	0.004	84	169	169				
3	Control	27299	WT	M	-0.006	-0.003	-0.004	14	29	29				
4	Control	27860	WT	M	-0.003	0.003	0.000	50	100	100				
5	Control	27238	WT	F	-0.011	-0.005	-0.008	-15	-30	0				
6	Control	27250	WT	F	-0.008	-0.012	-0.010	-32	-63	0				
7	Control	27303	WT	F	-0.019	-0.009	-0.014	-62	-125	0				
8	Control	27881	WT	F	-0.004	-0.007	-0.005	9	17	17				
											**80**	**117**	**41**	**8**
9	Control	27769	CYT HOM	M	-0.002	-0.007	-0.004	61	122	122				
10	Control	27770	CYT HOM	M	-0.010	-0.003	-0.006	47	94	94				
11	Control	27771	CYT HOM	M	-0.020	-0.014	-0.017	-22	-45	0				
12	Control	27887	CYT HOM	M	-0.009	-0.011	-0.010	26	52	52				
13	Control	28045	CYT HOM	F	-0.018	-0.023	-0.021	-45	-90	0				
14	Control	27962	CYT HOM	F	-0.023	-0.015	-0.019	-33	-66	0				
15	Control	27368	CYT HOM	F	-0.024	-0.029	-0.026	-84	-167	0				
16	Control	27902	CYT HOM	F	-0.021	-0.030	-0.026	-78	-156	0				
											**33**	**50**	**18**	**8**
17	PHZ Day 4	27325	WT	M	0.264	0.267	0.266	1,823	9,116	9,116				
18	PHZ Day 4	27795	WT	M	0.383	0.358	0.370	2,003	55,127	55,127				
19	PHZ Day 4	27798	WT	M	0.414	0.399	0.407	2,277	60,595	60,595				
20	PHZ Day 4	27816	WT	M	0.695	0.563	0.629	3,957	94,205	94,205				
21	PHZ Day 4	27313	WT	F	0.278	0.281	0.279	1,912	9,558	9,558				
22	PHZ Day 4	27857	WT	F	0.710	0.722	0.716	4,618	107,417	107,417				
23	PHZ Day 4	27819	WT	F	1.015	1.032	1.023	6,765	33,826	33,826				
24	PHZ Day 4	27933	WT	F	0.309	0.333	0.321	1,632	47,693	47,693				
											**52,192**	**35,707**	**12,624**	**8**
25	PHZ Day 4	27706	CYT HOM	M	0.160	0.168	0.164	1,161	5,803	5,803				
26	PHZ Day 4	27833	CYT HOM	M	0.269	0.282	0.276	1,887	9,437	9,437				
27	PHZ Day 4	27332	CYT HOM	M	0.252	0.274	0.263	1,807	9,035	9,035				
28	PHZ Day 4	27831	CYT HOM	M	0.117	0.119	0.118	96	16,972	16,972				
29	PHZ Day 4	27961	CYT HOM	F	0.379	0.383	0.381	2,086	56,783	56,783				
30	PHZ Day 4	27897	CYT HOM	F	0.202	0.213	0.207	1,441	7,206	7,206				
31	PHZ Day 4	27896	CYT HOM	F	0.342	0.357	0.350	2,371	11,853	11,853				
32	PHZ Day 4	27891	CYT HOM	F	0.170	0.180	0.175	523	25,526	25,526				
											**17,827**	**16,981**	**6,004**	**8**

Treatment	Number	Acss2 Geno	Gender	Day 0 Hct	Day 4 Hct	mCyc Ct	mAcss2 Ct		dCt	dCT of normalizer	ddCt	quantity
Control (harvest Day 4)	27221	WT	M	50.0	50.0	25.89	29.86		3.97	2.58	1.40	0.38
Control (harvest Day 4)	27244	WT	M	50.0	50.0	24.35	26.18		1.82	2.58	-0.76	1.69
Control (harvest Day 4)	27299	WT	M	50.0	50.0	23.75	24.85		1.09	2.58	-1.49	2.80
Control (harvest Day 4)	27860	WT	M	48.7	45.4	24.00	26.16		2.16	2.58	-0.42	1.33
Control (harvest Day 4)	27238	WT	F	50.0	50.0	24.11	26.63		2.52	2.58	-0.06	1.04
Control (harvest Day 4)	27250	WT	F	51.3	51.3	27.47	29.62		2.15	2.58	-0.43	1.34
Control (harvest Day 4)	27303	WT	F	48.7	48.7	24.13	26.05		1.91	2.58	-0.66	1.59
Control (harvest Day 4)	27881	WT	F	51.3	44.4	24.06	29.05		4.99	2.58	2.41	0.19
								**mean**	**2.58**			**1.30**
								**n**	**8**			**8**
								**STD**	**1.27**			**0.81**
								**SEM**	**0.45**			**0.29**
Control (harvest Day 4)	27769	CYT HOM	M	50.0	50.0	23.89	26.46		2.56	2.58	-0.02	1.01
Control (harvest Day 4)	27770	CYT HOM	M	51.1	51.2	24.11	26.84		2.73	2.58	0.15	0.90
Control (harvest Day 4)	27771	CYT HOM	M	50.0	48.5	23.75	26.68		2.93	2.58	0.35	0.78
Control (harvest Day 4)	27887	CYT HOM	M	48.5	45.9	23.67	26.30		2.63	2.58	0.05	0.97
Control (harvest Day 4)	28045	CYT HOM	F	46.9	46.0	23.29	27.66		4.37	2.58	1.79	0.29
Control (harvest Day 4)	27962	CYT HOM	F	48.5	50.2	23.48	27.36		3.89	2.58	1.31	0.40
Control (harvest Day 4)	27368	CYT HOM	F	47.5	48.3	24.50	28.07		3.57	2.58	0.99	0.50
Control (harvest Day 4)	27902	CYT HOM	F	46.6	48.3	24.05	28.49		4.44	2.58	1.86	0.28
								**mean**	**3.39**			**0.64**
								**n**	**8**			**8**
								**STD**	**0.78**			**0.31**
								**SEM**	**0.28**			**0.11**

	WT	CYT HOM
**AVG**	1.30	0.64
**SEM**	0.29	0.11

Normalized	WT	CYT HOM
**AVG**	1.00	0.50
**SEM**	0.22	0.08
**n**	8	8

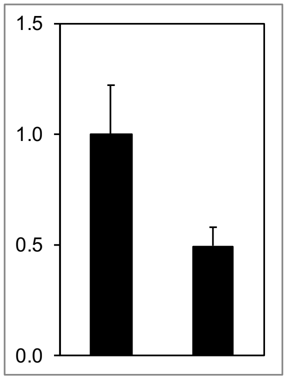

Treatment	Number	Acss2 Geno	Gender	Day 0 Hct	Day 4 Hct	mCyc Ct	mAcss2 Ct		dCt	dCT of normalizer	ddCt	quantity
Control (harvest Day 4)	27221	WT	M	50.0	50.0	21.54	25.03		3.49	3.28	0.21	0.86
Control (harvest Day 4)	27244	WT	M	50.0	50.0	21.23	24.46		3.22	3.28	-0.05	1.04
Control (harvest Day 4)	27299	WT	M	50.0	50.0	20.73	23.46		2.74	3.28	-0.54	1.46
Control (harvest Day 4)	27860	WT	M	48.7	45.4	21.48	24.12		2.63	3.28	-0.65	1.57
Control (harvest Day 4)	27238	WT	F	50.0	50.0	21.85	24.95		3.09	3.28	-0.18	1.14
Control (harvest Day 4)	27250	WT	F	51.3	51.3	21.55	24.68		3.13	3.28	-0.15	1.11
Control (harvest Day 4)	27303	WT	F	48.7	48.7	21.34	24.01		2.67	3.28	-0.61	1.53
Control (harvest Day 4)	27881	WT	F	51.3	44.4	22.86	28.12		5.26	3.28	1.98	0.25
								**mean**	**3.28**			**1.12**
								**n**	**8**			**8**
								**STD**	**0.86**			**0.43**
								**SEM**	**0.30**			**0.15**
Control (harvest Day 4)	27769	CYT HOM	M	50.0	50.0	21.00	26.03		5.04	3.28	1.76	0.30
Control (harvest Day 4)	27770	CYT HOM	M	51.1	51.2	21.02	25.24		4.22	3.28	0.94	0.52
Control (harvest Day 4)	27771	CYT HOM	M	50.0	48.5	21.28	26.09		4.82	3.28	1.54	0.34
Control (harvest Day 4)	27887	CYT HOM	M	48.5	45.9	21.29	26.42		5.13	3.28	1.85	0.28
Control (harvest Day 4)	28045	CYT HOM	F	46.9	46.0	20.74	25.54		4.81	3.28	1.53	0.35
Control (harvest Day 4)	27962	CYT HOM	F	48.5	50.2	20.92	23.54		2.62	3.28	-0.66	1.58
Control (harvest Day 4)	27368	CYT HOM	F	47.5	48.3	21.23	26.71		5.48	3.28	2.20	0.22
Control (harvest Day 4)	27902	CYT HOM	F	46.6	48.3	21.54	25.62		4.08	3.28	0.80	0.57
								**mean**	**4.52**			**0.52**
								**n**	**8**			**8**
								**STD**	**0.90**			**0.44**
								**SEM**	**0.32**			**0.16**

	WT	CYT HOM
**AVG**	1.12	0.52
**SEM**	0.15	0.16

Normalized	WT	CYT HOM
**AVG**	1.00	0.46
**SEM**	0.14	0.14
**n**	8	8

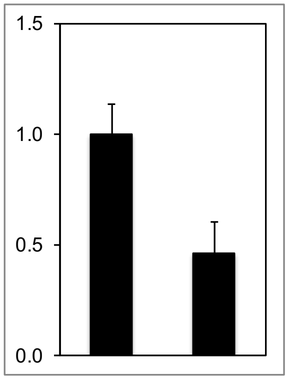

Sample	Gene	mCyc Ct	mCyc Ct Mean	mCyc Ct StdDev
WT	Cyclo	20.02	19.76	0.37
WT	Cyclo	19.93		
WT	Cyclo	19.34		
CYT HOM	Cyclo	20.26	19.85	0.44
CYT HOM	Cyclo	19.90		
CYT HOM	Cyclo	19.39		

Sample	Gene	mCyc Ct (Mean)	mAcss2 Ct	mAcss2 Ct Mean	mAcss2 Ct StdDev
WT	mAcss2	19.76	26.74	26.39	0.46
WT	mAcss2	19.76	26.56		
WT	mAcss2	19.76	25.86		
CYT HOM	mAcss2	19.85	28.05	27.57	0.54
CYT HOM	mAcss2	19.85	27.67		
CYT HOM	mAcss2	19.85	26.99		

Sample	mCyc Ct (Mean)	mAcss2 Ct		dCt	dCT of normalizer	ddCt	quantity
WT	19.76	26.74		6.97	6.62	0.35	0.78
WT	19.76	26.56		6.80	6.62	0.18	0.89
WT	19.76	25.86		6.10	6.62	-0.53	1.44
			**mean**	**6.62**			**1.04**
			**n**	**3**			**3**
			**STD**	**0.46**			**0.35**
			**SEM**	**0.27**			**0.20**
CYT HOM	19.85	28.05		8.20	6.62	1.58	0.33
CYT HOM	19.85	27.67		7.82	6.62	1.20	0.44
CYT HOM	19.85	26.99		7.14	6.62	0.52	0.70
			**mean**	**7.72**			**0.49**
			**n**	**3**			**3**
			**STD**	**0.54**			**0.19**
			**SEM**	**0.31**			**0.11**

	WT	CYT HOM
**AVG**	1.04	0.49
**SD**	0.35	0.19

Normalized	WT	CYT HOM
**AVG**	1.00	0.47
**SD**	0.34	0.18
**n**	8	8

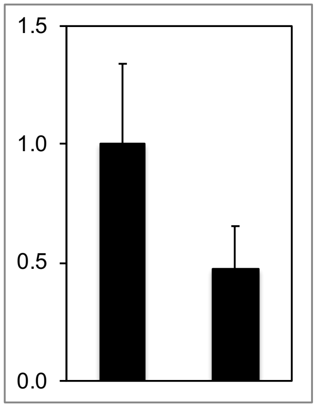

Supplement: S1 Table — The source data for the chart data presented in Figs 2A, 2B, 2C, 3C, 4C, and 6C are presented in separate worksheets within an Excel file. (PDF) [file pone.0225105.s001.pdf]
