# Supplementary material for: A substitution mutation in a conserved domain of mammalian acetate-dependent acetyl CoA synthetase 2 results in destabilized protein and impaired HIF-2 signaling
Source: PLoS One. 2019 Nov 14;14(11):e0225105. doi: 10.1371/journal.pone.0225105 (PMC6855420; doi:10.1371/journal.pone.0225105)

A Acss2 modeling predictions: Surface representations

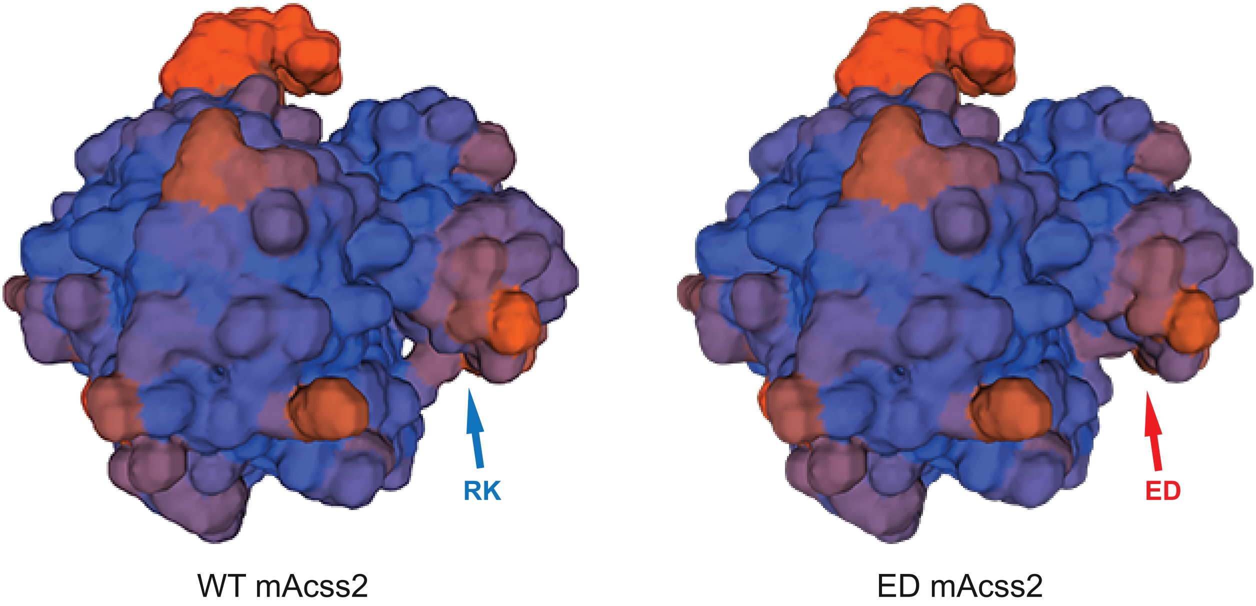

B Acss2 modeling predictions: Tube representations

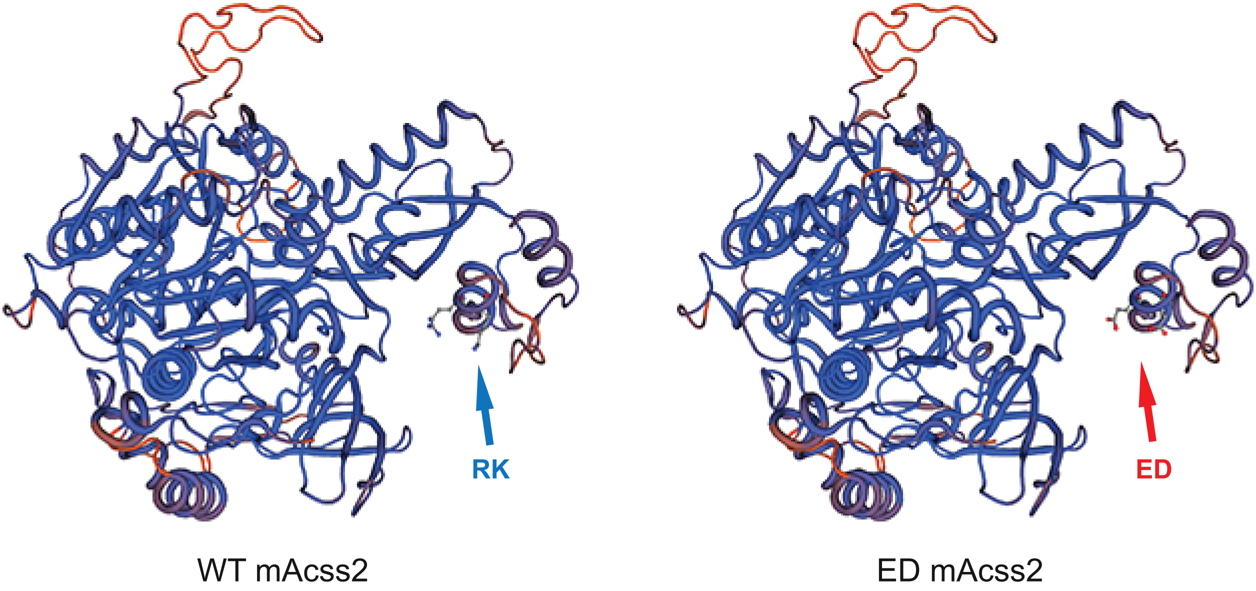

Supplement: S1 Fig — (A) The amino acid sequences of WT (NCBI Reference Sequence NP_062785.2) and ED mouse Acss2 (mAcss2) protein was used in SWISS-MODEL for modeling predictions. Surface representations of WT and ED mAcss2 proteins are shown. The location of the parental amino acid residues (RK) in WT mAcss2 is indicated by a blue arrow and the substitution amino acid residues (ED) in ED mAcss2 is indicated by a red arrow. Loss of an electrostatic bridge is seen in ED mAcss2 protein compared with WT mAcss2 protein. (B) Tube representations of WT and ED mAcss2 proteins with parental residues in WT mAcss2 protein (RK) and substitution residues in ED mAcss2 protein (ED) indicated as above and also by ball and stick figures. (PDF) [file pone.0225105.s002.pdf]
